# A systematic review of the determinants of vaccine hesitancy in the UK: Implications for future TB vaccine adoption

**DOI:** 10.1186/s12889-026-28299-9

**Published:** 2026-06-30

**Authors:** Annabel Kennard, Aysenur Kılıç, Dana Alsugeir, Yohhei Hamada, Molebogeng X. Rangaka

**Affiliations:** 1 Institute of Global Health, University College London, London, UK; 2 Institute of Health Informatics & UCL School of Pharmacy, University College London, London, UK; 3 College of Pharmacy, Imam Abdulrahman Bin Faisal University, Dammam, Saudi Arabia; 4 Division of Epidemiology and Biostatistics & CIDRI-AFRICA, University of Cape Town, Cape Town, South Africa

**Keywords:** Tuberculosis, Vaccine hesitancy, Adoption, System, Individual

## Abstract

**Background:**

Tuberculosis (TB) is a deadly yet vaccine-preventable disease mostly affecting adolescents and adults. Novel TB vaccines are now emerging, particularly for these populations; however, vaccine hesitancy, characterised by delays, doubts or refusal to seek or accept vaccines, poses a challenge for sufficient adoption once these vaccines become available. Thus, this review systematically explored system- and individual-level determinants of vaccine hesitancy among adults in the UK, interpreting implications for the adoption of novel TB vaccines.

**Methods:**

Five databases systematically searched for studies from 2020 – 2024 reporting qualitative outcomes on determinants of vaccine hesitancy among people living in the UK. Thematic analysis mapped extracted data onto a framework combining the Strategic Advisory Group of Experts (SAGE) model to explore system-level factors (e.g., vaccine-specific influences) and the Perceptions and Practicalities Approach (PaPA) to explore individual-level factors (e.g., perceptual influences) of vaccine hesitancy. The Mixed Methods Appraisal Tool was used to assess study quality.

**Results:**

29 qualitative and 21 mixed methods studies; 87.5% investigated hesitancy to COVID-19 vaccines. All SAGE themes were identified at the system level, most notably vaccine novelty, the media environment and health system experiences. At individual-level, all PaPA themes emerged, with vaccine-specific beliefs and knowledge most frequently identified. Overall, themes varied across the lifespan, with adolescents influenced mainly by media and social norms, and older adults by safety concerns and trust in health professionals.

**Conclusions:**

Vaccine hesitancy determinants interacted and were compounded in vulnerable subgroups, while post-pandemic disruptions to TB services may undermine adoption of novel TB vaccines. Addressing these challenges requires multi-level strategies centred on trusted, culturally sensitive communication. Delivery through routine, school and community-based programs aligns with evidence from high-incidence settings and highlights the need for coordinated action by policymakers and healthcare providers.

**Supplementary Information:**

The online version contains supplementary material available at https://doi.org/10.1186/s12889-026-28299-9.

Tuberculosis (TB) represents a paradox of the 21st century: although it is preventable and mostly curable, it remains the deadliest infectious disease in the world [1]. In 2023, 10.8 million people developed TB, marking the highest rates recorded since 1993, when the WHO declared TB a global emergency [1]. Vaccines can play a key role in controlling TB by stopping it’s spread [2]. However, the only available TB vaccine is more than a century old, Bacillus Calmette–Guérin (BCG) [2]. BCG is mostly effective for infants and children, it has poor efficacy and adverse effects in late adolescent and adult populations [3]. Considering TB mostly affects late adolescent and adult populations [4], we urgently need safe and effective TB vaccines to enhance global TB prevention [5].

In recent years, several promising TB vaccine candidates for adolescents and adults, such as M72-AS01e, have entered large-scale Phase III trials assessing their effectiveness in high-incidence settings [6]. These vaccines are expected to prevent approximately 37.2 to 76 million cases by 2050 [7]. Despite these breakthroughs, research shows that the availability of vaccines does not guarantee that people will get vaccinated [8]. Vaccine hesitancy, the delay in acceptance or refusal to vaccinate despite availability of vaccination services, is among the top ten global health threats of the century [9]. It reduces the effectiveness of our tools [10]. For example, low uptake of the measles vaccine led to multiple outbreaks in 2019, increasing global cases by 30% in a single year and causing a resurgence in countries close to eliminating measles, such as the UK [10]. Therefore, it is crucial to understand the factors that influence vaccine hesitancy, especially among vulnerable groups such as migrant communities [11]. This understanding is essential to ensure the successful rollout of new TB vaccines and avoid the setbacks seen with malaria vaccines, where despite availability, low uptake delayed progress by nearly a decade [12, 13].

WHO Strategic Advisory Group of Experts on Immunisation (SAGE) indicates that vaccine hesitancy is “*complex and context-specific*,* varying across time*,* place*,* and vaccines*” [14] and maps out a framework to understand the drivers of vaccine hesitancy across systems to inform policy [15]. However, the SAGE framework is limited to specify person-centred drivers, which are essential for clinical practice [15]. Thus, we propose combining the SAGE framework with the Perceptions and Practicalities Approach (PaPA) [16] to ensure a holistic representation of both individual- and system-level drivers. Although PaPA was developed to understand medication adherence, it can be adapted to address vaccine hesitancy by placing the individual at the centre, focusing on their perceived necessity, concerns, and practical barriers to vaccination [16, 17]. By combining the SAGE framework with the PaPA, we aim to provide a holistic, person-centred framework that captures the drivers of vaccine hesitancy at both system and individual levels among adolescents and young adults in the UK. This framework is intended to inform the introduction of novel TB vaccines in key populations in low TB incidence settings.

The COVID-19 pandemic disrupted TB prevention and service uptake contributed to rising cases; TB notification rates in England increased by 11% in 2023, the largest annual rise since 2000, threatening progress toward *‘End TB’* targets [18, 19]. In the UK, 83% of TB services reported being adversely affected by the pandemic, with reductions in face-to-face consultations, latent TB screening, and clinical capacity further limiting service uptake [20]. To avoid similar barriers during the rollout of new TB vaccines, it is vital to learn from the post-pandemic context as research indicates that the COVID 19 pandemic fundamentally restructured public attitudes toward vaccination in the UK, and growing evidence suggests that hesitancy generated during this period has spilled over into broader vaccine scepticism [21–23]. Further, the emerging TB vaccines mostly targeting adolescents and adults marks a critical juncture in global TB control; however, the only available TB vaccine (BCG) mostly targets infants and children usually as part of routine vaccination programmes [3, 24]. Therefore, it is essential for us to learn from the post-COVID 19 context where adolescents and adults’ views towards vaccination explored. Yet to date, no review has systematically mapped these determinants among the adolescent and adult populations in the UK, who represent both the primary TB-vulnerable groups and the intended recipients of emerging TB vaccines. Thus, this review aims to identify key system- and individual-level factors that influence vaccine uptake across adolescents and adults in the UK, to inform effective public health strategies for TB vaccine implementation locally and beyond.

## Methods

### Search strategy

Searches were conducted on December 2nd 2024 in five databases; Medline (Ovid version), Global Health (Ovid version), Web of Science (Core collection), EMBASE and PsychINFO. Search terms and adapted search strategies were defined and reviewed. Three search concepts were identified; ‘United Kingdom’, ‘Vaccines’, and ‘Attitude & Behavioural’, narrowed to specific search terms. Boolean operators (‘AND’ & ‘OR’) and filters for time (2020 - present) and English language were applied. A detailed search strategy for each database is available in Appendix.1.

The protocol of this qualitative systematic review is registered (PROSPERO: CRD42024601756), and follows PRISMA 2020 guidance (Appendix 2) [25]. It was later amended to include all age groups over 12 years old.

### Inclusion and exclusion criteria

Studies published after 31 January 2020, enrolling UK residents or reporting UK-specific findings in multi-national studies and examining vaccine hesitancy where individuals made their own vaccination decisions, were included. No restrictions were placed on vaccine type; however, studies focusing exclusively on infant or child vaccination were excluded based on population, as these groups fall outside the TB-vulnerable age groups this review aimed to address. Excluded were studies published before this date, those focused on non-UK populations, or where others made vaccination decisions (e.g., caregivers). Conference abstracts and letters to the editor were also excluded.

### Data extraction

Two reviewers (AK & DA) independently conducted the literature search, data extraction, and coding, overseen by an experienced reviewer (AKi). A pre-defined strategy (Appendix 1) was applied to database searches, with additional grey literature reviewed separately. Results were imported into Zotero [26] for duplicate removal, followed by a second check in Rayyan [27] and final manual screening. Initial title and abstract screening followed by full-text screening by using a predetermined inclusion/exclusion screening table, with reasons for exclusion recorded. Each record was screened independently by two reviewers at both title and abstract and full-text stages, using a predetermined inclusion/exclusion screening table, with reasons for exclusion recorded. Reviewers’ agreement was assessed using Cohen’s Kappa, with disagreements resolved through discussion.

### Risk of bias

Two experienced independent reviewers (DA & AKi) assessed the quality of the included papers by using the Mixed Methods Appraisal Tool (MMAT) [28] and 10% the included papers were randomly selected to calibrate between reviewers.

### Data analysis & synthesis

This review employed a mixed-methods systematic review design, combining descriptive synthesis and qualitative deductive thematic analysis guided by the conceptual framework (described below) to extract and analyse data. Data on study characteristics, design, vaccine types and key findings were extracted from the included papers. Study characteristics selection followed Cochrane Collaboration guidelines for synthesising qualitative evidence [29], omitting outcomes as per Thomas & Harden’s (2008) [30] guidance for thematic analysis. For thematic analysis, data (results, participant quotes) was line-by-line coded manually into Excel by two reviewers (AK & DA), reviewed by another experienced reviewer (AKi) to minimise reporting bias and enhance interpretation of findings. Study characteristics and quality assessment were tabulated. Codes were sorted into predefined themes, and subthemes inductively generated, capturing specific contexts. Theme frequencies were counted once per paper to maintain data integrity without fragmenting participant responses [31, 32]. All results were tabulated. Representative quotes demonstrated key themes.

### Conceptual framework

Combining SAGE and PaPA to capture system- and individual-level determinants of vaccine hesitancy, SAGE model’s broad ‘individual and group influences’ category was reorganised to align with PaPA’s person-centred focus. Irrelevant factors, such as cost (BCG vaccines are free for those eligible in the UK) and dexterity (specific to medication), were excluded to tailor the PaPA framework to the research question. To explore possible causes of heterogeneity across results, as well as the importance of age on vaccine hesitancy toward TB vaccines, we also performed an age-based subgroup analysis. By using our conceptual framework to map vaccine hesitancy across the lifespan, we analysed theme frequencies across 3 age categories: adolescence (12–18 years), adulthood (18 to 64 years) and older adulthood (65 + years).

## Results

**Fig. 1:**
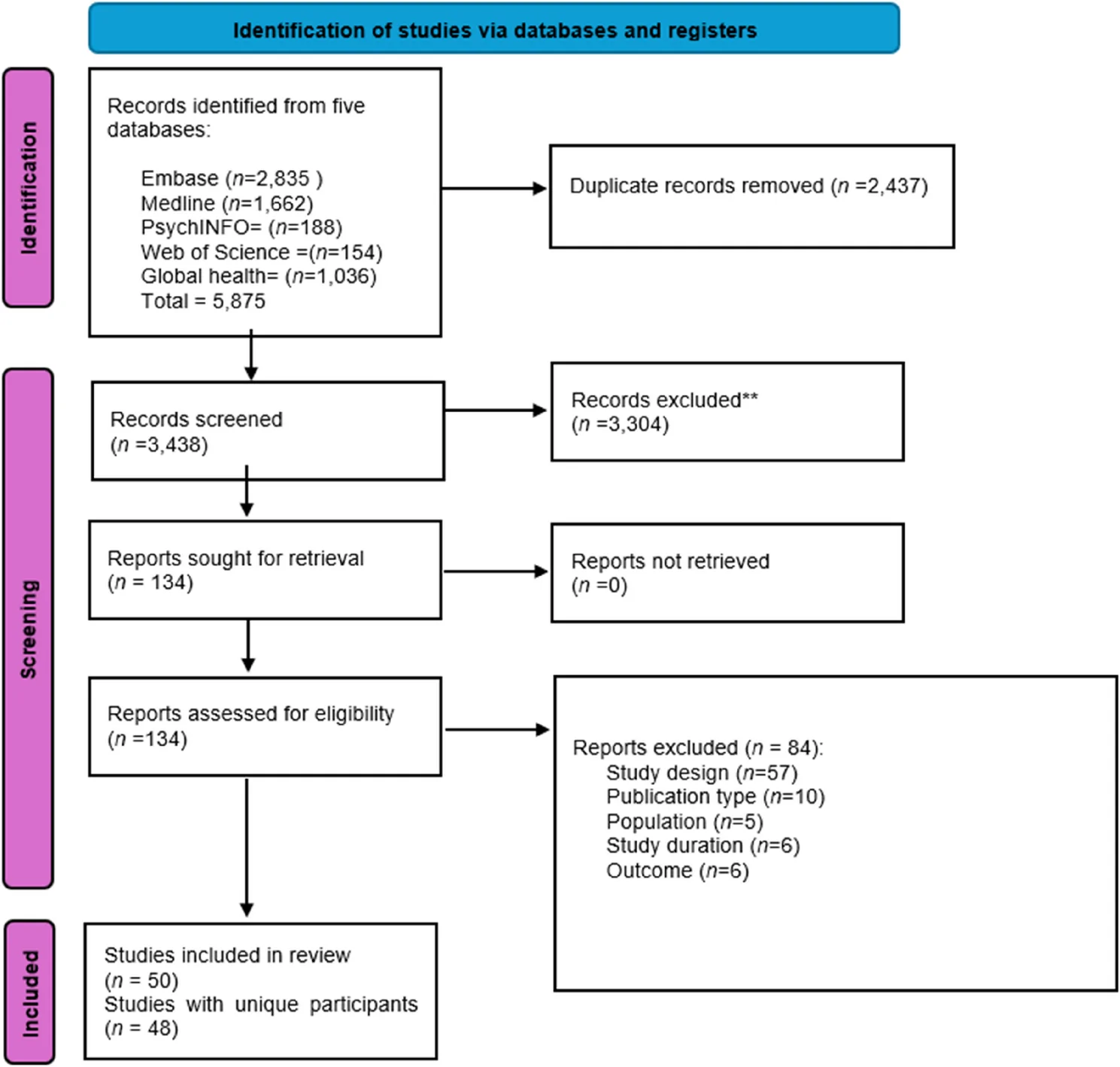
PRISMA flow diagram for study selection criteria

The initial search identified 5,875 studies (Fig. 1). After removing duplicates, 3,438 abstracts were screened, excluding 3,304. Of 134 full-text articles reviewed, 50 were included, with 48 having unique participants. Both papers were included despite sharing participants, as they reported different qualitative analyses and contributed distinct findings. Reviewers had substantial agreement and any disagreements among them were resolved through discussion.

Around 13,891 people who took part in qualitative (*n* = 29) and mixed‑methods (*n* = 21) studies, with sample sizes ranging from 10 to 4,353 participants (aged 12 to 80+), were included in this review. Few studies focused on young adults (10–24 years, *n* = 3), with only two including adolescents. Five examined pregnant women, two university students, and eight migrant, social, or ethnic minority groups. Most (*n* = 42) investigated COVID-19; others covered meningitis (*n* = 3), influenza (*n* = 3), HPV (*n* = 2), Td/IPV (*n* = 2), MMR (*n* = 2), pneumococcal (*n* = 2), pertussis, shingles, Mpox, routine maternal vaccines, and general vaccine beliefs (each, *n* = 1). Further details on population, vaccine type, study design, and setting are in Appendix 3.

### Quality assessment

Most qualitative and mixed methods studies were ranked as high quality (Appendix 4). Reasons for low quality in qualitative studies were having an unclear research question or an unclear interpretation of results. Common methodological concerns for mixed methods included small, homogenous samples, not accounting for confounding variables in non-randomised quantitative analysis and insufficient discussion of the researcher–participant relationship.

### Vaccine hesitancy framework

**Fig. 2:**
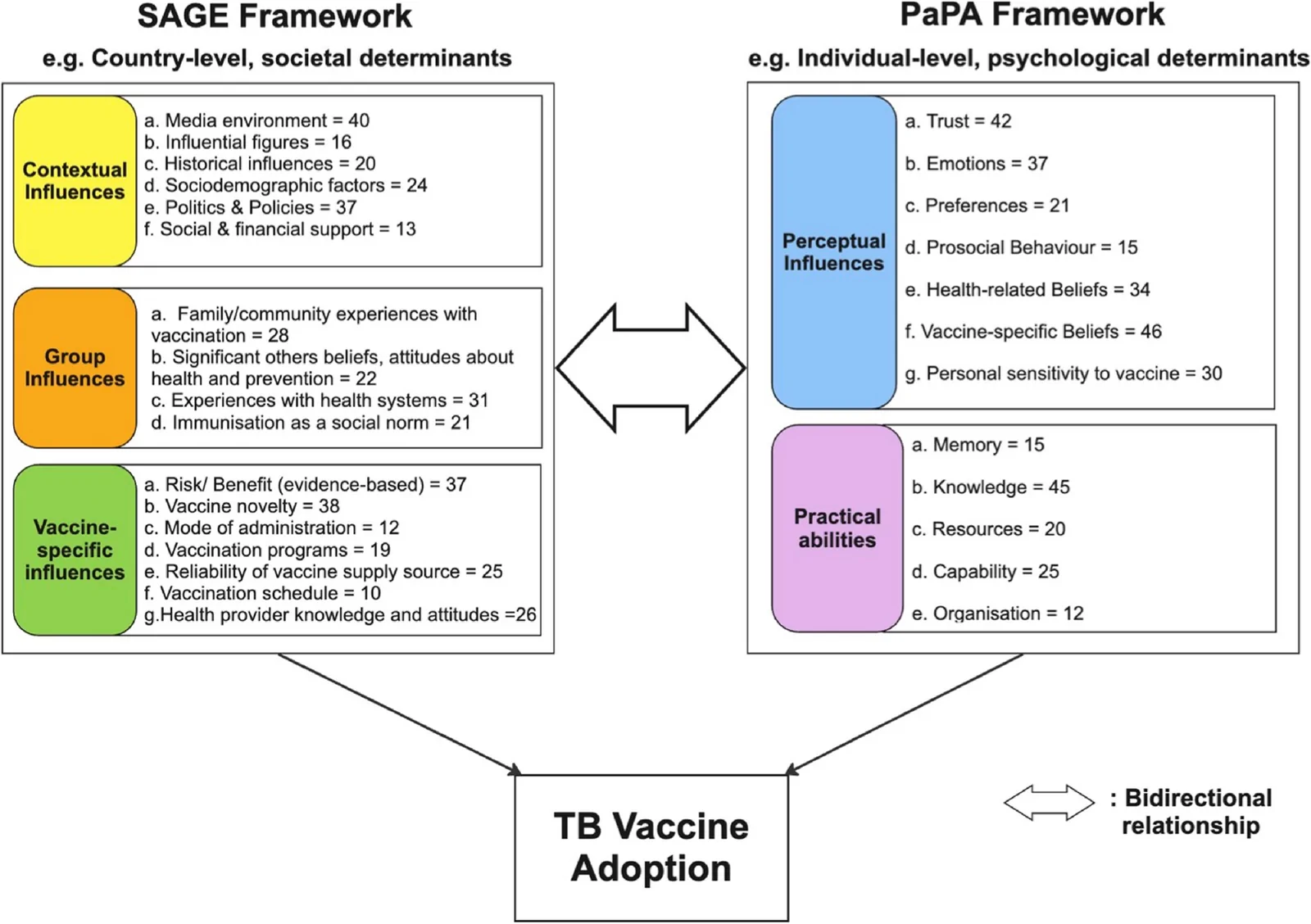
An integrated Framework for Vaccine Hesitancy among People in the UK towards a Novel TB Vaccine [14–16]

Results are reported for SAGE and PaPA frameworks (Fig. 2). Multiple themes across SAGE and PaPA frameworks often arose from the same data codes, represented by the bidirectional relationship between frameworks in Fig. 2. Representative quotes supporting each theme and subtheme are provided in Appendix 5.

Mapping vaccine hesitancy onto the SAGE framework. All predefined themes: contextual, group, and vaccine-specific, were identified across 50 studies. Key factors shaping hesitancy included media environment (*n* = 40), health system experiences (*n* = 31), and vaccine novelty (*n* = 38).

*Contextual influences.* Media environment (*n* = 40), followed by and linked to, political (*n* = 37) and sociodemographic factors (*n* = 24) *contributed to* vaccine hesitancy. Traditional media influences tied to political factors, due to *“unclear and generic”* vaccine communication during the pandemic [33]. People distrusted government-affiliated media, like the BBC, calling for transparent, science-based communication. Inconsistent health messaging was viewed indicative of governmental uncertainty [34]. They were sceptical of mass vaccination, believing economic interests were prioritised, increasing distrust [35]. Additionally, historical scandals like the Thalidomide tragedy and the MMR scare, left legacies of distrust in authoritative advice [36, 37].

Social media emerged as a distinct but linked barrier, amplifying mistrust and confusion already fuelled by political and traditional media narratives. Participants reported repeated exposure to alarming content such as infertility, blood clots, microchip implantation, or targeted harm to specific communities, most often via WhatsApp, Facebook, Instagram, and YouTube [38–40]. Contradictory narratives, often rooted in partial truths or outdated findings, gained traction when shared by trusted contacts or individuals presenting as credible professionals [41, 42]. Rapid circulation of flawed or later-disproven studies further shaped vaccine beliefs, with limited public ability to judge research quality allowing misinformation to persist [40].

Others described distress from emotive but false videos designed to scare-monger for views:

Contradictions between online narratives and official guidance undermined confidence in the government and pharmaceutical companies, particularly among ethnic minority and younger participants reliant on social media and word-of-mouth [33, 40, 43]. Polarised “echo chambers” reinforced scepticism [44], while removal of anti-vaccination content was sometimes interpreted as censorship, heightening suspicion [45]. Even when not the primary source, social media was reported to amplify fears and entrench mistrust, destabilising public health messaging [46–48]. Social media also further spread disease stigma, such as discouraging Mpox patients from presenting to health services [49].

Sociodemographic factors like ethnicity, religion, immigration and socioeconomic status intersected with political and media distrust, reinforcing vaccine hesitancy. Ethnic minority and migrant participants felt excluded from policy design, calling for culturally relevant public health campaigns [50, 51]. Racist experiences in younger female South and East Asian participants left reports it *“more safer to stay at home”* than seek vaccination [37]. Religious concerns, like vaccines containing porcine products among Muslim communities and HPV vaccine morality in Catholic groups, also drove hesitancy [43, 52]. Furthermore, systemic inequities in social and financial support (*n* = 13) worsened hesitancy in lower-income participants [35, 37, 53]. Together distrust, misinformation and marginalisation fuelled vaccine hesitancy among people in the UK.

*Group Influences.* ‘Experiences with health systems’ (*n* = 31) most frequently affected vaccine hesitancy, alongside community experiences (*n* = 28) and significant others health and prevention beliefs (*n* = 22). Social pressure (*n* = 21) was also prevalent.

During the pandemic, participants expressed frustration with the healthcare system’s poor proactive outreach, limiting vaccine information and appointment access [49, 51, 54]. Young undocumented migrants were particularly disengaged, fearing immigration status exposure [55]. Disengagement, combined with vaccine supply shortages, undermined the system’s credibility to manage vaccination effectively, increasing hesitancy [36, 45, 50]. Despite health system issues, providers like GPs and nurses were trusted figures in participants’ decision-making processes, yet provided inconsistent advice [56, 57]. Their role in providing clear vaccine information was important, especially to adolescents, suggesting healthcare providers deliver health education and vaccination within more familiar, school-settings, particularly for HPV vaccines [52, 58].

Family and social networks also shaped people’s vaccine attitudes [38, 43, 59]. Parental beliefs particularly influenced adolescents [48, 52]. Further, community leaders were *“go-to trusted sources”* over authorities, although participants noted their absence from public health messaging [44, 50, 51]. Additionally, anecdotes on adverse vaccine experiences circulated within social circles, reported to be more influential than government advice by some participants [59–62]. In many cases (*n* = 21), social pressure reported led participants to either vaccinate despite personal doubts, reinforcing regret and long-term mistrust, or avoid vaccination altogether [37, 44, 63].

*Vaccine-specific influences.* Vaccine novelty (*n* = 38) was the largest influence, followed by risk-benefit analyses (*n* = 37). Although less frequent, mode of administration (*n* = 12), vaccine programs (*n* = 19), and healthcare providers’ vaccine-specific attitudes (*n* = 26) also shaped hesitancy. Vaccine-specific influences overlapped with PaPA, described below.

Across most studies, participants expressed concern with the rapid development of COVID-19 vaccines, raising doubts over clinical trial rigor and limited long-term data. Safety concerns, especially around fertility, led to adoption of a “*wait and see*” approach, delaying vaccination until more information became available [61, 64, 65]. Novelty concerns intersected with risk-benefit analyses [34, 63]. Participants questioned the vaccine’s effectiveness to prevent transmission, especially when vaccinated individuals *“still contracted COVID-19 or were hospitalised”* [48, 58]. Severe side effect reports, like blood clotting and allergic reactions linked to AstraZeneca, heightened concerns, exacerbated by perceived insufficient clinical trial numbers to ensure safety [66]. Frequent booster doses were interpreted as limited efficacy to some, resulting in refusal of vaccination [36, 56, 67].

Some participants criticised the poor management of vaccination programs, mainly COVID-19 (*n* = 19), perceiving slow, inequitable vaccine distribution, limited transparency and inaccessible vaccination sites [38, 50, 52, 60]. Overexposure to mass-vaccination campaigns, as with the COVID-19 drive, disengaged some altogether [49, 55]. Distrust in vaccine supply reliability (*n* = 25) and the pharmaceutical industry, added to concerns about insufficient testing on diverse populations and handling issues for Moderna and Pfizer vaccines [39, 43, 50, 68]. Furthermore, scientist disagreements and trusted healthcare provider attitudes (*n* = 26) translated into conflicting, inconsistent guidance, causing confusion and hesitancy [33, 56, 59, 60].

Mapping vaccine hesitancy onto the PaPA framework. All themes were identified, significance varied, and subthemes overlapped. Vaccine-specific beliefs (*n* = 46) were the most common perceptual influence, while limited knowledge (*n* = 45) primarily drove practical vaccine hesitancy in the UK.

*Perceptual Influences*. Vaccine-specific concerns (*n* = 46) about safety and necessity were central to exploring vaccine hesitancy among young people. Common perceptual influences included emotional responses (*n* = 37), health-related beliefs (*n* = 34) and distrust (*n* = 42). Prosocial behaviour (*n* = 15) and personal sensitivity (*n* = 30) also contributed.

Health-related and vaccine-specific beliefs were central to hesitancy in many adults and young people, who perceived low risk to diseases like COVID-19 and flu, given their age and health, and deemed vaccination unnecessary [62, 66]. Low necessity often coupled with safety concerns, fearing short-term side effects and unknown long-term risks [69]. One participant even remarked *“I could die”* [32], while another mentioned: “*Risks associated with negative side effects from the vaccine outweigh the risks associated with the virus*” [66]. Safety concerns translated into emotional reactions (*n* = 37) like anxiety toward long-term risks, and more immediate fears like needle phobia, especially in younger individuals [58, 62]. Young migrants also revealed trauma-related injection fears from prior vaccinations [55, 65]. Further, participants felt frustrated toward government and media pressures to vaccinate, while some reported guilt and ethical conflict [33, 37, 68].

As explored in the SAGE framework, deep distrust (*n* = 42) in government, healthcare services and pharmaceutical companies drove hesitancy, exacerbated by excessive and inconsistent health promotion [37, 38, 43]. A minority of participants held extreme conspiracy beliefs, viewing vaccination as a coercive measure to *“curb the population”* or *“bribery”*, eroding trust [34, 37, 44, 50].

Interestingly, prosocial behaviour (*n* = 15) drove hesitancy, as participants believed they were protecting and prioritising vulnerable populations by rejecting vaccination, as opposed to herd immunity [43, 48, 59]. Hesitant individuals preferred (*n* = 21) visible evidence of vaccine safety and fewer *“burdensome”* boosters [53, 62, 67]. Those with personal sensitivities (*n* = 30) also expressed vaccine-related concerns, requesting personalised pre-vaccination screening [54, 59, 60, 70].

*Practical influences.* Practical abilities affected both vaccine hesitancy and access, where poor access to knowledge (*n* = 45) and resources (*n* = 20) dissuaded hesitant individuals.

Across most studies, limited vaccine-specific knowledge (*n* = 45) was evident, compounded by contextual factors like inconsistent COVID 19 messaging [71]. Safety concerns over insufficient research on long-term risks for vulnerable groups amplified doubts, as one participant explained: *“There is no actual facts on how safe this vaccine is during pregnancy.”* [56]. Some participants even limited their own knowledge exposure by actively avoiding removing vaccine content from their feeds as a form of self-protection due to distrust or emotional discomfort [59].

Studies interviewing young migrants and mothers, found participants unaware of available routine vaccinations in adulthood (e.g. meningitis, polio, etc.), or misunderstood their purpose, reducing uptake [61, 65]. For adolescents, poor awareness of eligibility and autonomy to self-consent to HPV vaccines left many uninformed about their necessity or benefits, intersecting with perceptual influences [52]. Language barriers further limited access to reliable information, deepening mistrust in underserved groups [55].

Poor resource access (*n* = 20) at both system and individual levels increased vaccine hesitancy. Underfunded outreach left many uninformed on vaccine availability, while complex booking system, appointment shortages and short-notice or inconvenient timings undermined trust and heightened frustration [49, 53, 58]. Capability barriers (*n* = 25), including physical or health constraints, scheduling or eligibility conflicts, language difficulties and challenges navigating the health system further limited engagement with vaccination services [55]. Financial barriers such as travel costs, also restricted access [37, 53] and poor recall of past vaccinations reduced confidence in the process [48]. Participants suggested that health education and vaccine provision in schools and universities would improve uptake [52, 62]. These personal and systemic challenges were reported to diminish both people’s ability and desire to engage with vaccination services [40, 41, 51].

### Vaccine hesitancy across the lifespan: Adolescence, adulthood and older adulthood

Three major life stages, adolescence (12 to 18 years), adulthood (18 to 64 years) and older adulthood (65 + years), were examined separately to understand the vaccine hesitancy across the lifespan (Appendix 6). Vaccine hesitancy was mostly explored among adulthood (*n* = 19), followed by adolescence (*n* = 2) and older adulthood (*n* = 1).

Since adulthood was the most examined life stage for vaccine hesitancy, all themes were represented when analyses were limited to this stage. While all core themes from the SAGE and PaPA frameworks were represented in both adolescence and older adulthood, their subthemes differed slightly. For example, although SAGE contextual influences were reported in both groups, adolescents showed influences related to sociodemographic factors, politics and policies, alongside pronounced effects of the media environment, whereas media exposure was the only contextual influence reported for older adults [65, 72]. Adolescents’ hesitancy was shaped by media-driven misinformation and political distrust [48, 52]. Their doubts often reflected the rapid spread of vaccine rumours on social media and a sense of being poorly addressed by formal health communication [41, 73]. Concerns regarding personal autonomy, influenced by peer dynamics, also shaped group and practical influences [52]. In contrast, older adults’ hesitancy centred around vaccine safety, side-effects, and fear of the unknown, often informed by previous negative experiences, misinformation encountered interpersonally, and a preference for natural immunity [72]. Whereas adolescents were influenced by social norms and social media, older adults relied more heavily on health professionals, family history and system-level accessibility [48, 72].

Together these findings suggest that vaccine hesitancy is shaped by age-specific contexts: adolescents navigate a socially and digitally mediated information landscape, while older adults draw heavily on health system trust, experiential knowledge and personal vulnerability.

## Discussion

This review identified a complex interplay of system- and individual-level factors driving vaccine hesitancy among adolescents and adults in the UK, with implications for novel TB vaccine adoption. The dominant system-level determinants were- media distrust, negative healthcare experiences, and vaccine novelty concerns, while safety fears, low perceived necessity, and limited knowledge shaped individual-level hesitancy. These determinants were especially pronounced in underserved groups including migrants, pregnant women, and young people. Although most included studies examined COVID-19 hesitancy, these determinants are not necessarily COVID-19 specific; they characterise a post-pandemic hesitancy environment, given that vaccine novelty was the most frequently identified vaccine-specific influence (*n* = 38). Novel TB vaccines such as M72-AS01e face precisely this profile: an unfamiliar product, targeting an under-discussed disease, in a population whose trust in new vaccines has been materially disrupted [6, 74]. Evidence of COVID-19 hesitancy spillover further underscores the urgency of proactive, tailored, multi-level strategies to support TB vaccine uptake, particularly among vulnerable subgroups [21–23].

At the system level, vaccine hesitancy is shaped by media narratives, political dynamics, and healthcare system experiences, often amplified by social networks. Distrust in government-affiliated media and inconsistent health messaging may erode public confidence, particularly among younger populations who rely heavily on social media for health information [39, 40]. This aligns with global concerns about insufficient political commitment to address vaccine-related stigma and misinformation [75]. In high-income, low-incidence settings, limited public and provider awareness of diseases like TB may further compound hesitancy [76, 77]. Comparative studies suggest that personal sensitivity to health risks can either drive engagement or avoidance, depending on the perceived reliability of healthcare systems [78–80]. Science-informed communication and improved healthcare engagement are important for supporting the uptake of novel TB vaccines in these contexts [81].

Social networks, including family, peers, and community leaders, play a key role in shaping vaccine attitudes [38, 50, 59]. This aligns with broader evidence from high-income settings showing that trust in local figures can influence vaccine decisions, especially among adolescents and underserved groups [82–86]. Community-based and school-based programs have shown promise in improving vaccine uptake by embedding trusted health education in familiar environments [6, 85]. Early engagement through routine services and education settings may help counter misinformation and foster positive attitudes toward new TB vaccines [85].

At the individual level, vaccine hesitancy is often driven by safety concerns, emotional responses to novelty, and limited trust in unfamiliar vaccines [85]. Familiar options, such as BCG revaccination, are sometimes preferred over newer candidates due to perceived risks, even when efficacy is higher [6]. These concerns are particularly evident among individuals with perceived sensitivities or comorbidities, who reported a need for personalised pre-vaccination assessment to confirm their suitability for vaccination and reduce fears of adverse effects [59, 60, 87, 88]. Similar concerns about multi-dose regimens and side effects have been observed with COVID-19 boosters [89]. Routine delivery is generally favoured over mass campaigns, which some view as overwhelming or stigmatising [55]. Embedding TB vaccines into routine immunisation schedules may therefore support acceptability and uptake in high-risk groups [74].

Practical barriers such as inconvenient clinic hours, travel distances, and limited access to reliable information also hinder vaccine acceptance [90]. These challenges are particularly pronounced among socially disadvantaged populations, including migrants and young adults [91]. Addressing these barriers through accessible, equitable, and trusted healthcare services would help support successful TB vaccine implementation [73, 85, 92].

### Strengths and limitations

Our review encompassed a large number of studies with diverse methodologies and patient populations, many of which demonstrated robust research designs, allowing for a comprehensive understanding of vaccine hesitancy and its implications for TB vaccine adoption. However, a subset of studies exhibited methodological limitations, including unclear research questions and inadequate control for confounding variables in observational designs. The complexity and context-specificity of vaccine hesitancy complicated data synthesis across studies, making broad generalisations challenging [9]. Despite critiques over limited flexibility, deductive approaches complemented this study’s large data set, regarded as gold-standard for coding reliability, objectivity and accuracy [93]. Using a comprehensive conceptual framework to predefine themes and guide data extraction enhanced the clarity and specificity of findings [93]. Most included studies focused on COVID‑19 vaccines, with no studies examining TB‑specific vaccines. While some determinants of vaccine hesitancy may be theoretically transferable across contexts, these findings should be interpreted cautiously to avoid over‑generalisation. Vaccine acceptance can vary substantially depending on the specific vaccine profile, and future TB vaccines may involve distinct considerations that differ from those associated with COVID‑19. As TB‑specific research in low‑incidence settings emerges, future reviews should revisit this question. Lastly, the review could not be updated beyond 2024 due to the substantial volume of new literature and resource constraints.

### Practice and Policy Recommendations for Adoption of a New TB vaccine

**Fig. 3:**
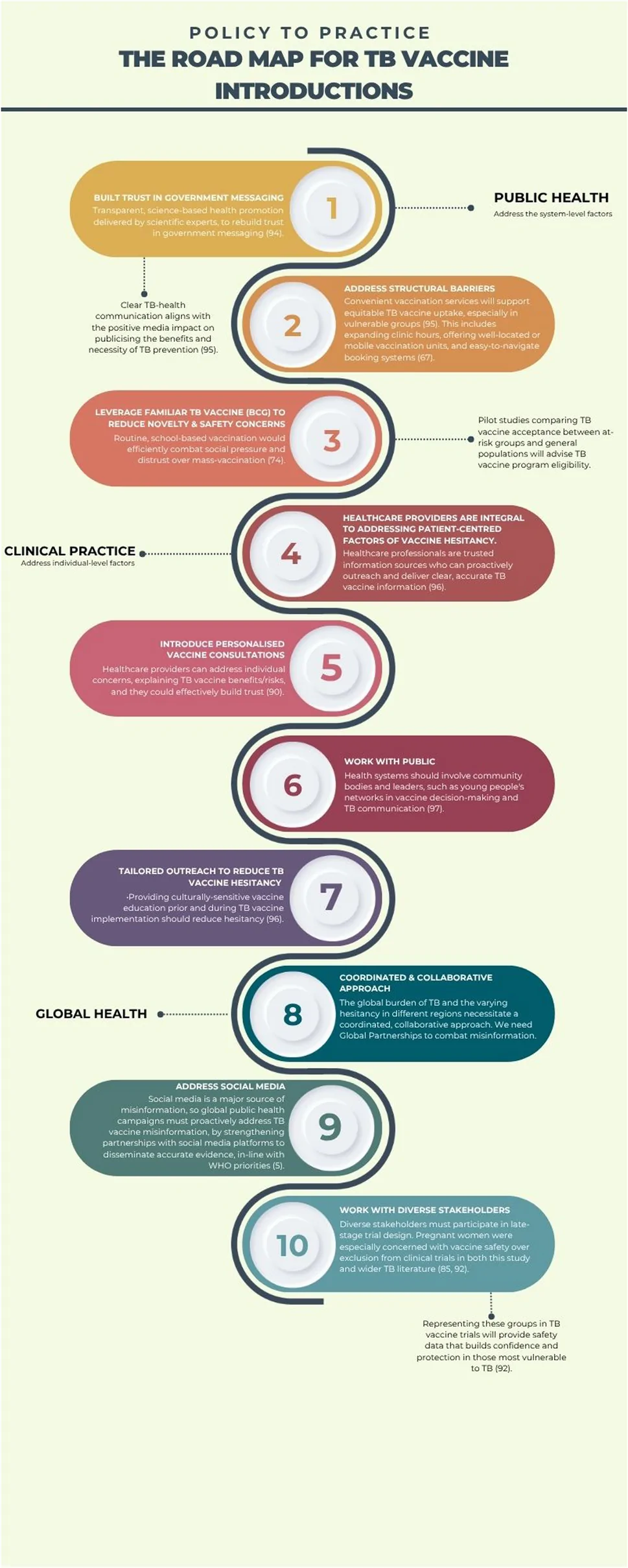
Policy To Practice: The Road Map For TB Vaccine Introduction. Conceptual framework synthesised from existing literature [5, 74, 85, 90, 92, 94–97]

Multi-level strategies are needed to support TB vaccine uptake among adolescents and young adults in high-income, low TB incidence settings. Recommendations span system-level (public health) and individual-level (clinical) approaches, aligned with review objectives summarised in an infographic (Fig. 3).

### Conclusion

As vaccination expands across the lifespan, new vaccines, including for TB, are likely to target adolescents and adults. This review highlights key barriers to uptake in high-income, low-incidence TB settings, including systemic distrust and individual concerns around safety, necessity, and access. These insights are vital for supporting the transition of TB vaccines from trials to real-world use. Transparent communication, stronger healthcare engagement, and inclusive outreach will be critical to build trust. Priority research needs include UK-based qualitative studies examining TB-specific vaccine beliefs in high-risk populations, alongside longitudinal work tracking how post-pandemic hesitancy evolves as novel TB vaccines approach licensure, to inform policy and support successful vaccine rollout aligned with global End-TB goals.

## Supplementary Information

Supplementary Material 1.

## Data Availability

All data supporting the findings are derived from previously published articles, all data supporting the findings of this study are available within the paper and its Supplementary Information.

## Abbreviations

TB: Tuberculosis
WHO: World Health Organization
SAGE: WHO Strategic Advisory Group of Experts on Immunisation
PaPA: Perceptions and Practicalities Approach
