# Supplementary material for: A systematic review of the determinants of vaccine hesitancy in the UK: Implications for future TB vaccine adoption
Source: BMC Public Health. 2026 Jun 30;26:2831. doi: 10.1186/s12889-026-28299-9 (PMC13644071; doi:10.1186/s12889-026-28299-9)
Supplement: Supplementary file 1 [file 12889_2026_28299_MOESM1_ESM.docx]

# Appendix 1

***Search Strategy***

| Database | Search Strategy | Filters |
| --- | --- | --- |
| MEDLINE (Ovid) | (((((vaccine[MeSH Terms]) OR (Vacc* OR immunis* OR immuniz* OR inoculation)) AND (Attitude to health[MeSH Terms])) OR (hesitan* OR uptake OR "up-take" OR trust* OR distrust* OR accept* OR refusal OR "anti-vaccination" OR willing* OR perception* OR belief* OR view* OR perspective* OR attitude* OR concern* OR decision* OR refus* OR decline[MeSH Terms]) ) AND (UK OR United Kingdom OR England OR Wales OR Scotland OR Northern Ireland OR GB OR Great Britain) | Date limit: Jan 2020–present |
| EMBASE (Ovid) | 1. exp vaccine/ OR exp immunization/ OR vaccin*.ti,ab. OR immunis*.ti,ab. OR immuniz*.ti,ab. OR inoculat*.ti,ab. 2. exp health attitude/ OR exp health belief/ OR exp vaccine hesitancy/ OR hesitan*.ti,ab. OR uptake.ti,ab. OR trust*.ti,ab. OR distrust*.ti,ab. OR accept*.ti,ab. OR refusal.ti,ab. OR "anti-vaccination".ti,ab. OR belief*.ti,ab. OR attitude*.ti,ab. OR concern*.ti,ab. OR decision*.ti,ab. 3. (UK OR "United Kingdom" OR England OR Wales OR Scotland OR "Northern Ireland" OR "Great Britain" OR GB).ti,ab. 4. 1 AND 2 AND 3 | Date limit: Jan 2020–present |
| PsycINFO (Ovid) | 1. exp Vaccines/ OR exp Immunization/ OR vaccin*.ti,ab. OR immuniz*.ti,ab. OR immunis*.ti,ab. OR inoculat*.ti,ab. 2. exp Attitudes/ OR exp Health Attitudes/ OR exp Vaccine Hesitancy/ OR hesitan*.ti,ab. OR uptake.ti,ab. OR trust*.ti,ab. OR accept*.ti,ab. OR refus*.ti,ab. OR "anti-vaccination".ti,ab. OR belief*.ti,ab. OR attitude*.ti,ab. OR perception*.ti,ab. OR concern*.ti,ab. OR decision*.ti,ab. 3. (UK OR "United Kingdom" OR England OR Wales OR Scotland OR "Northern Ireland" OR "Great Britain" OR GB).ti,ab. 4. 1 AND 2 AND 3 | Date limit: Jan 2020–present |
| Global Health (Ovid) | 1. vaccin*.tw. OR immunis*.tw. OR immuniz*.tw. OR inoculat*.tw. 2. hesitan*.tw. OR uptake.tw. OR trust*.tw. OR accept*.tw. OR refusal.tw. OR "anti-vaccination".tw. OR belief*.tw. OR attitude*.tw. OR perception*.tw. OR concern*.tw. OR decision*.tw. 3. (UK OR "United Kingdom" OR England OR Wales OR Scotland OR "Northern Ireland" OR "Great Britain" OR GB).tw. 4. 1 AND 2 AND 3 5. Limit 4 to yr="2020 -Current" | Date limit: Jan 2020–present |
| Web of Science | TS=(vaccin* OR immunis* OR immuniz* OR inoculat*) AND TS=(hesitan* OR uptake OR trust* OR accept* OR refusal OR "anti-vaccination" OR belief* OR attitude* OR perception* OR concern* OR decision*) AND TS=(UK OR "United Kingdom" OR England OR Wales OR Scotland OR "Northern Ireland" OR "Great Britain" OR GB) Refine by: Publication Years = (2020–present) | Date limit: Jan 2020–present |

# Appendix 2

***PRISMA Checklist***

| **Section and Topic** | **Item #** | **Checklist item** | **Location where item is reported** |
| --- | --- | --- | --- |
| **TITLE** | | |  |
| Title | 1 | Identify the report as a systematic review. | p. 1, Title |
| **ABSTRACT** | | |  |
| Abstract | 2 | See the PRISMA 2020 for Abstracts checklist. | p. 2, Abstract |
| **INTRODUCTION** | | |  |
| Rationale | 3 | Describe the rationale for the review in the context of existing knowledge. | p. 3-5,  Introduction |
| Objectives | 4 | Provide an explicit statement of the objective(s) or question(s) the review addresses. | p. 4-5, Introduction |
| **METHODS** | | |  |
| Eligibility criteria | 5 | Specify the inclusion and exclusion criteria for the review and how studies were grouped for the syntheses. | p. 6,  Methods |
| Information sources | 6 | Specify all databases, registers, websites, organisations, reference lists and other sources searched or consulted to identify studies. Specify the date when each source was last searched or consulted. | p. 6,  Methods |
| Search strategy | 7 | Present the full search strategies for all databases, registers and websites, including any filters and limits used. | p. 6,  Methods |
| Selection process | 8 | Specify the methods used to decide whether a study met the inclusion criteria of the review, including how many reviewers screened each record and each report retrieved, whether they worked independently, and if applicable, details of automation tools used in the process. | p. 6-7,  Methods |
| Data collection process | 9 | Specify the methods used to collect data from reports, including how many reviewers collected data from each report, whether they worked independently, any processes for obtaining or confirming data from study investigators, and if applicable, details of automation tools used in the process. | p. 6-7,  Methods |
| Data items | 10a | List and define all outcomes for which data were sought. Specify whether all results that were compatible with each outcome domain in each study were sought (e.g. for all measures, time points, analyses), and if not, the methods used to decide which results to collect. | p. 6-8,  Methods |
|  | 10b | List and define all other variables for which data were sought (e.g. participant and intervention characteristics, funding sources). Describe any assumptions made about any missing or unclear information. | p. 6-8,  Methods |
| Study risk of bias assessment | 11 | Specify the methods used to assess risk of bias in the included studies, including details of the tool(s) used, how many reviewers assessed each study and whether they worked independently, and if applicable, details of automation tools used in the process. | p. 7,  Methods |
| Effect measures | 12 | Specify for each outcome the effect measure(s) (e.g. risk ratio, mean difference) used in the synthesis or presentation of results. | N/A |
| Synthesis methods | 13a | Describe the processes used to decide which studies were eligible for each synthesis (e.g. tabulating the study intervention characteristics and comparing against the planned groups for each synthesis (item #5)). | p. 7-8,  Methods |
|  | 13b | Describe any methods required to prepare the data for presentation or synthesis, such as handling of missing summary statistics, or data conversions. | NA |
|  | 13c | Describe any methods used to tabulate or visually display results of individual studies and syntheses. | p. 7-8,  Methods |
|  | 13d | Describe any methods used to synthesize results and provide a rationale for the choice(s). If meta-analysis was performed, describe the model(s), method(s) to identify the presence and extent of statistical heterogeneity, and software package(s) used. | p. 8,  Methods |
|  | 13e | Describe any methods used to explore possible causes of heterogeneity among study results (e.g. subgroup analysis, meta-regression). | p. 8, Methods |
|  | 13f | Describe any sensitivity analyses conducted to assess robustness of the synthesized results. | N/A |
| Reporting bias assessment | 14 | Describe any methods used to assess risk of bias due to missing results in a synthesis (arising from reporting biases). | N/A |
| Certainty assessment | 15 | Describe any methods used to assess certainty (or confidence) in the body of evidence for an outcome. | N/A |
| **RESULTS** | | |  |
| Study selection | 16a | Describe the results of the search and selection process, from the number of records identified in the search to the number of studies included in the review, ideally using a flow diagram. | p. 9,  Results |
|  | 16b | Cite studies that might appear to meet the inclusion criteria, but which were excluded, and explain why they were excluded. | N/A  Results |
| Study characteristics | 17 | Cite each included study and present its characteristics. | p. 10,  Results &  Appendix 3 |
| Risk of bias in studies | 18 | Present assessments of risk of bias for each included study. | p. 10,  Results.  Appendix 3 |
| Results of individual studies | 19 | For all outcomes, present, for each study: (a) summary statistics for each group (where appropriate) and (b) an effect estimate and its precision (e.g. confidence/credible interval), ideally using structured tables or plots. | N/A |
| Results of syntheses | 20a | For each synthesis, briefly summarise the characteristics and risk of bias among contributing studies. | p.11, Results.  Appendix 4 |
|  | 20b | Present results of all statistical syntheses conducted. If meta-analysis was done, present for each the summary estimate and its precision (e.g. confidence/credible interval) and measures of statistical heterogeneity. If comparing groups, describe the direction of the effect. | N/A |
|  | 20c | Present results of all investigations of possible causes of heterogeneity among study results. | p. 9-18, Appendix 3 & 4 |
|  | 20d | Present results of all sensitivity analyses conducted to assess the robustness of the synthesized results. | N/A |
| Reporting biases | 21 | Present assessments of risk of bias due to missing results (arising from reporting biases) for each synthesis assessed. | N/A |
| Certainty of evidence | 22 | Present assessments of certainty (or confidence) in the body of evidence for each outcome assessed. | N/A |
| **DISCUSSION** | | |  |
| Discussion | 23a | Provide a general interpretation of the results in the context of other evidence. | p. 19-20,  Discussion |
|  | 23b | Discuss any limitations of the evidence included in the review. | p. 21,  Discussion |
|  | 23c | Discuss any limitations of the review processes used. | p. 21,  Discussion |
|  | 23d | Discuss implications of the results for practice, policy, and future research. | p. 21-22,  Discussion |
| **OTHER INFORMATION** | | |  |
| Registration and protocol | 24a | Provide registration information for the review, including register name and registration number, or state that the review was not registered. | p. 6,  Methods |
|  | 24b | Indicate where the review protocol can be accessed, or state that a protocol was not prepared. | p. 6,  Methods |
|  | 24c | Describe and explain any amendments to information provided at registration or in the protocol. | p. 6,  Methods |
| Support | 25 | Describe sources of financial or non-financial support for the review, and the role of the funders or sponsors in the review. | p. 23,  Declarations |
| Competing interests | 26 | Declare any competing interests of review authors. | p. 23,  Declarations |
| Availability of data, code and other materials | 27 | Report which of the following are publicly available and where they can be found: template data collection forms; data extracted from included studies; data used for all analyses; analytic code; any other materials used in the review. | p. 22-23,  Declarations.  Appendix 1, 2 & 4. |

*From:*  Page MJ, McKenzie JE, Bossuyt PM, Boutron I, Hoffmann TC, Mulrow CD, et al. The PRISMA 2020 statement: an updated guideline for reporting systematic reviews. BMJ 2021;372:n71. doi: 10.1136/bmj.n71. This work is licensed under CC BY 4.0. To view a copy of this license, visit <https://creativecommons.org/licenses/by/4.0/>

# Appendix 3

***Descriptives of Included Studies***

| Author (Year) | Study Location | Sample Characteristics | Study Design | Aims and Measurements | Data Analysis | Vaccine Type(s) | Results | Limitations |
| --- | --- | --- | --- | --- | --- | --- | --- | --- |
| Qualitative designs (*n* = 29) | | | | | | | | |
| Anderson et al. (2021) | England (Bristol) | 31 pregnant women  Age range: 24-48 years | Semi-structured interviews | To explore: i) how the COVID-19 pandemic affected pregnant women's access to and attitudes toward routine maternal vaccines; and ii) their views on testing COVID-19 vaccines during pregnancy and personal willingness to participate. | Thematic analysis | Routine maternal vaccines | Participants valued maternal vaccines more due to the pandemic; but, had safety concerns about appointments. Most viewed the new COVID-19 vaccine as riskier than the virus itself. | - a homogenous, pro-vaccine sample may underestimate hesitancy.  - over representation of university staff could skew views.  - early-pandemic interviews may not reflect current attitudes.  - limited vaccine access issues reported, reducing insights on distributions. |
| Biesty et al. (2024) | England (Liverpool) | 26 national and regional key informants involved in mpox response and adults at high-risk of mpox  Age range: 25-61 years | Semi-structured interviews & participatory workshops | Exploring stigma and health-seeking behaviour in the recent mpox outbreak. | Deductive framework analysis | mpox | Stigma around mpox was common, but its effect on health-seeking behaviour was reduced through efforts by the LGBTQ+ community, sexual health clinics, and the third sector. However, key informants felt these were undermined by under-resourced, poorly communicated, and evidence-lacking public health responses. | - Liverpool-focused study limits generalisability.  - Underrepresentation of younger, student, and closeted MSM.  - Incentive-based recruitment may introduce bias.  - No data on participants’ previous mpox diagnoses.  - Unmeasured factors like perceived vaccine scarcity not accounted for. |
| Crawshaw et al. (2023) | England (London: Hackney) | 32 Congolese adult migrants  *Estimated** age range:  25 – 80 years | In-depth interviews & co-design workshops | Co-designing a culturally tailored intervention to boost COVID-19 vaccine uptake among Congolese migrants in London, by identifying vaccination barriers, facilitators, and information needs. | Thematic analysis mapped onto the TDF and COM-B models | COVID-19 vaccines | The study identified 14 barriers, such as safety, effectiveness, necessity, communication, and distrust, and 10 facilitators including access, medical advice, risk awareness, social influence, trust, belief in research, and protective intent | - no intervention was implemented or evaluated due to time and budget constraints.  - academics led the study and framework, reinforcing power imbalances and potentially overlooking systemic issues like racism.  - funding limitations highlight the ned for larger samples, longer timelines, and stronger community partnerships. |
| Crawshaw et al. (2024a) | England (London: Hackney) | 32 Congolese adult migrants  *Estimated* age range:  25 – 80 years | Peer-led, semi-structured interviews | Exploring factors shaping COVID-19 vaccine decisions among Congolese migrants, including migration experiences, healthcare access, and vaccination perceptions. | Reflexive thematic analysis | COVID-19 and routine vaccines | Participants' concerns about COVID-19 vaccination stemmed from safety, effectiveness, past racial discrimination and exploitation, with feelings of being ignored and government messaging seen as coercive. | - focusing on a small sample for a specific community makes results not generalisable to other marginalised communities coming from different contexts. |
| Dasgupta et al. (2024) | UK | 45 adults  Age range:  23 – 70 years | Semi-structured in-depth interviews | Exploring lived experiences and views of women, partners, HCPs, and policymakers on offering the COVID-19 vaccine during pregnancy. | Thematic analysis | COVID-19 vaccines | Themes include mistrust of drugs during pregnancy, prior positive experiences, concerns over misinformation and unclear vaccine guidance, and behaviours shaped by participants' experiences and available information. | - underrepresentation of healthcare professionals and policymakers. |
| Deal et al. (2021) | UK | 32 migrants  *Estimated* age range: 30 – 38 years | Semi-structured in-depth interviews | Exploring views on COVID-19 vaccine access, with input on action points and solutions to improve uptake in marginalised migrant groups. | Thematic analysis | COVID-19 vaccines | Barriers included unclear access points, distrust in healthcare, and pandemic-related neglect. Language barriers, lack of GP registration, and fear of immigration checks were common. Hesitancy stemmed from misinformation, safety concerns, and poor communication. Trusted clinics, anonymity, and clear multilingual information were key to boosting uptake. | - lack of geographical representation from across the UK,  - may have a bias towards those who have a higher comprehension of English. |
| Deal et al. (2024) | England (London:  Hackney) | 71 migrant adults  Age range:  18 – 60+ years | Semi-structured in-depth interviews | Exploring adult migrants' experiences with catch-up vaccination to inform strategies that strengthen related policy and practice. | Thematic framework analysis | MMR, Tetanus, Diphtheria, Polio, HPV, Meningitis | Catch-up vaccines were rarely offered but generally welcomed when explained. Concerns included side effects, access, and COVID-19 fatigue. Trust in healthcare shaped decisions, though past NHS experiences and discrimination led to some mistrust. | - limited geographical representation, lack of diversity in nationalities and age groups.  - the potential influence of ongoing COVID-19 vaccination campaigns on participants' views.  - no survey tools were used to assess moral intuitions. |
| Denford et al. (2022) | UK | 70 adults  *Sample 1:* 35 adults received one dose of the vaccine  Age range:  30 – 49 years  *Sample 2:* 35 adults who did not vaccinate  Age range:  18 – 29 years | Semi-structured interviews | Exploring factors affecting COVID-19 vaccine uptake and views on initiatives targeting vaccine-hesitant individuals. | Thematic analysis | COVID-19 vaccines | Participants vaccinated for safety and to avoid restrictions, though concerns over efficacy, safety, and access remained. Uptake was shaped by age, health, trust, and scientific literacy, with vaccine passes viewed as both incentive and pressure. | - potential bias from missing voices of non-computer literate individuals, combining age groups and vaccination status.  - reliance on phone interviews which may have influenced responses, and inability to verify self-reported vaccination status. |
| Dennis et al. (2021) | England (Northwest) | 10 care home employers  Age range:  25 – 61 years | Semi-structured in-depth interviews | Exploring: i) reasons for care home staff declining COVID-19 vaccines; ii) factors that may increase uptake among previously unvaccinated staff; iii) attitudes towards mandatory vaccination policies. | Framework analysis | COVID-19 vaccines | Eight themes: risk perception, vaccine effectiveness and concerns, mistrust in authorities, uptake facilitators, views on mandates, negative care work experiences, and communication issues. | - limited representation to the Northwest care home employees and may not be generalisable to other care home settings in England. |
| Eberhardt et al. (2023) | UK | 95 Black and Asian adults  Age range:  18 – 70+ years | Online engagement platform discussions | Exploring factors affecting vaccine hesitancy in Black and Asian adults to address concerns about vaccination. | Thematic analysis | COVID-19 vaccines | Mixed views on COVID-19 vaccination included concerns over targeted messaging for Black and Asian communities, rollout confusion, hesitancy, and reliance on trusted local networks for information. | - lack of diverse participant characteristics, such as religion or gender limiting generalisability of findings to diverse populations. |
| Fisher et al. (2021) | England (Southwest) | 21 adolescents  Age range:  12 – 17 years | Semi-structured interviews | Exploring adolescents’ experiences on vaccination during the COVID-19 pandemic. | Thematic analysis | COVID-19 vaccines | Participants’ concerns about COVID-19 vaccination stemmed from issues of safety, effectiveness, and past racial discrimination, accompanied by feelings of being ignored and perceptions of government messaging as coercive. | - no data on participants’ socioeconomic status or ethnicity, limiting representativeness.  - broad study design restricted deep exploration of specific issues. |
| Flood et al. (2024) | Northern Ireland | 34 adolescents  Age range:  15 – 16 years | Focus groups | exploring 15–16-year-olds’ views on current HPV education and its influence on vaccine intention and sexual health decisions. | Directed content analysis mapped to the COM-B model | HPV vaccine | Students felt their HPV knowledge was poor and supported mandatory education at 15–16. Barriers included parental views, school ethos, religion, and lack of awareness of self-consent rights. Addressing these could improve sexual health, awareness, and vaccine uptake. | - some TDF domains were excluded from focus groups.  - sample skewed toward middle/higher socioeconomic backgrounds and female students.  - potential selection bias from participating schools and teachers.  - limited generalisability beyond Northern Ireland due to its unique ethnic and religious context. |
| Friedrich et al. (2024) | England | 20 care home staff  Age range: *Unclear* | Semi-structured interviews | understanding the factors influencing uptake of COVID-19 vaccination in care home staff | Thematic analysis | COVID-19 vaccines | Uptake was driven by a desire for protection, supportive environments, and hopes of normality. Concerns included rapid development, limited long-term data, and risks of side effects or allergic reactions. | - limited generalisability due to interviewing only vaccinated people. |
| Fuller et al. (2022) | England  (London, Brighton, & Sussex) | 20 immunosuppressed adults with IMIDs  Age range:  27 – 72 years | Semi-structured in-depth interviews | To explore barriers and facilitators to COVID-19, influenza, and pneumococcal vaccine uptake in immunosuppressed adults with immune-mediated inflammatory diseases (IMIDs). | Thematic analysis mapped onto the SAGE VH matrix | COVID-19, influenza, and pneumococcal vaccines | Vaccine uptake was hindered by concerns over IMID flare-ups, active disease, low perceived risk, and lack of HCP guidance. Facilitators included perceived risk, belief in benefits, and HCP advice. COVID-19 uptake was shaped by media, case rates, and visibility, while flu/pneumococcal uptake was influenced by eligibility awareness and direct invitations. | - self-selecting participants and a small sample size could introduce sampling bias. |
| Gillibrand et al. (2024) | England  (Greater Manchester) | 35 community residents, community leaders, and health and care system stakeholders  Age range:  18 – 80+ years | One-to-one interviews & focus groups | exploring public responses to the COVID-19 vaccination program, focusing on inequalities through perspectives of under-served communities and stakeholders. | Thematic framework analysis | Vaccines in general | Public responses to COVID-19 vaccination were shaped by institutional distrust and social inequalities, deepened by the pandemic’s disproportionate impact on minoritised groups, youth, and those with health conditions. | - small but diverse sample with limited generalisability.  - may not fully reflect experiences of all marginalised groups.  - captures attitudes at one pandemic stage; views may have shifted over time. |
| Gogoi et al. (2022) | UK | 164 HCPs  Age range:  32 – 53 years | One-to-one interviews & focus groups | understanding HCPs attitudes and perceptions towards the COVID-19 vaccines, and factors associated with vaccine decision making. | Content & thematic analysis | COVID-19 vaccines | Even though most HCPs were willing to accept vaccines, some had passive acceptance accompanied with hesitancy. | - limited generalisability to the users of digital technologies.  - selection bias towards people who are more willing to accept the vaccines. |
| Hicks et al. (2022) | UK | 14 adults who did not received or invited for COVID-19 vaccination  Age range: *Unclear* | Semi-structured interviews | Identifying what shape information literacy practice takes for people who are hesitant about the COVID-19 vaccines. | Inductive thematic analysis with constructivist grounded theory | COVID-19 vaccines | vaccine-hesitant individuals engage in five key information activities (listening to stories, reading the body, pooling information, confirming against expertise, and hedging) to navigate COVID-19 vaccine information, reflecting an agentic approach to information literacy in this context. | - online recruitment bias.  - convenience sampling.  - lack of demographic data. |
| Lockyer et al. (2021) | UK (Bradford) | 20 adults  Age range:  18 – 65+ years | In-depth interviews | Exploring attitudes towards COVID-19 vaccines. | Reflexive thematic analysis | COVID-19 vaccines | Hesitancy stemmed from safety concerns, negative stories, and personal knowledge. Confusion and mistrust during the pandemic reduced vaccine positivity. | - interviews limited to a single area,  - views reflect announcements before the efficacious COVID‐19 vaccines. |
| Lockyer et al. (2022) | UK  (Bradford) | 12 adults  Age range:  25 – 65+ years | In-depth interviews | Exploring change in attitudes towards the vaccines. | Reflexive thematic analysis | COVID-19 vaccines | Participants chose vaccination due to workplace risk, protecting loved ones, travel ease, and influence from close contacts. | - small sample size, - individuals unwilling to take the vaccine were unwilling to participate |
| Magee et al. (2022) | England (London) | 38 Black and South Asian adults from primary care settings  Age range:  19 – 82 years | Semi-structured interviews | Exploring COVID-19 vaccine hesitancy in primary care patients from South Asian and Black ethnicities. | Thematic analysis | COVID-19 vaccines | Concerns included rollout speed, mistrust in official info, and negative messaging; facilitators were healthcare discussions, social influence, and incentives. | - limited and varied ethnic group representation.  -possible mismatches between researchers and participants.  - social desirability bias affecting responses.  - selection bias due to language barriers.  - lack of longitudinal data on vaccine decision-making. |
| Naqvi et al. (2022) | UK | 12 vaccine hesitant adults from ethnic minorities  Age range: *Unclear* | Semi-structured interviews | Investigating factors behind vaccine hesitancy among UK ethnic minority groups, examining social media's influence, and identifying strategies to improve vaccine acceptance. | Conventional content analysis,  Framework method. | COVID-19 vaccines | Social media fuelled vaccine hesitancy through misinformation on side effects and infertility, while trusted non-social media sources encouraged uptake. Travel desires and positive public health messaging boosted vaccination, but a lack of trusted information increased hesitancy. | - limited sample size with incomplete ethnic minority representation.  - excluded non-English speakers.  - findings may not be specific to ethnic minority communities. |
| Ogueji & Okoloba (2022) | Only UK data included | 30 UK residents  Age range:  18 – 79 years | Qualitative design using online data collection forms | Examining drivers of COVID-19 vaccine acceptance and obstacles to uptake. | Thematic analysis | COVID-19 vaccines | Few participants were vaccinated availability was the main driver, with mild side effects. Hesitancy arose from safety concerns, perceived benefits, mistrust, vague messaging, and moral conflict. Barriers included access issues, low perceived risk, and minority status. Solutions involved clearer communication, endorsements, free access, incentives, and transparency | - limited generalisability of qualitative findings as a small number of vaccinated participants, restricting insights into vaccination drivers.  - selection bias due to social media-based snowball sampling, excluding digitally disconnected individuals. |
| Parsons et al. (2024) | England (Yorkshire, Humber & Sheffield) | 43 pregnant women  Age range:  18 – 44 years | Semi-structured interviews | Exploring how pregnant women feel about these vaccinations, and what factors influence the uptake of vaccinations onset of the Covid-19 pandemic. | Thematic analysis | Influenza, pertussis & COVID-19 vaccines | Internal factors and beliefs, vaccination related factors, external influences and Covid-19 and changing perceptions of the pandemic influenced the vaccine uptake. | - post-pandemic timing of interviews, which may have shaped responses, and isolating COVID-19 vaccination from routine vaccines despite potential overlap in public attitudes. |
| Patterson et al. (2023) | Northern Ireland | 13 new / expectant mothers with vaccine hesitancy  Age range:  18 – 45 years | Semi-structured interviews & focus groups | Understanding barriers and facilitators to COVID-19 vaccine uptake using the COM-B framework to make recommendations for public health interventions. | Semi-deductive reflexive thematic analysis | COVID-19 vaccines | Vaccine decisions were influenced by the need for clear, tailored information, social influences, and integration into antenatal care. While valuing health autonomy, participants faced internal conflict over long-term baby health concerns, with fear, lack of empathy, and guilt driving indecision. | - small sample size due to limited time and funding.  - underrepresentation of minority ethnic groups despite higher COVID-19 risk and lower vaccine uptake.  - need for further research to support targeted interventions for these populations. |
| Poduval et al. (2023) | England | 22 adults from ethnic minorities  Age range:  21 – 67 years | One-to-one interviews & focus groups | Improve the understanding of COVID-19 vaccine confidence and the structural factors influencing confidence in low-uptake ethnic minority groups in London and the surrounding areas. | Inductive reflexive  thematic analysis | COVID-19 vaccines | People from ethnic minority backgrounds base COVID-19 vaccine decisions on external factors (e.g. mistrust in political authority, healthcare neglect, structural racism) and internal reasoning (e.g. weighing vaccine risks, benefits, and development concerns). | - research focused on a single geographic area.  - timing may have influenced participant responses.  - small participant numbers within each ethnic group.  - language barriers may have affected data quality.  - interviewer ethnicity may have impacted participant openness. |
| Sides et al. (2022) | England & Wales | 100 adults from 19 self-identified ethnic groups  Age range:  30 – 39 years | One-to-one interviews & focus groups | Exploring attitudes and intentions towards COVID-19 vaccination and sources of COVID-19 information. | Inductive thematic analysis mapped into TDF | COVID-19 vaccines | Mistrust and safety concerns were common across ethnic groups, with vaccine decisions shaped by occupation, health, and social influences. Facilitators included hopes of normality, perceived risk, and access. Information sources varied by social and cultural context, with widespread concern over misinformation. | - possible selection bias in sample.  - limited representation of ethnic groups.  - non-English speakers excluded from study.  - data collected pre-vaccine rollout; attitudes may have evolved.  - researcher demographics may have influenced interpretation. |
| Van Bergen et al. (2022) | UK | 18 adults  Age range:  18 – 29 years | One-to-one interviews | Identifying the underlying beliefs regarding COVID-19 vaccinations among young adults in the UK. | Content analysis | COVID-19 vaccines | Intenders of vaccination and non-intenders shared similar beliefs overall, but non-intenders had greater concerns about side effects, particularly long-term risks like blood clotting and fertility issues, while both groups were largely influenced by their friends and family. | - cross-sectional design limits causal interference.  - self-reported data vulnerable to social desirability bias.  - vaccination intentions may change over time.  - dichotomised intent categories may oversimplify views.  - purposive sampling may not represent wider population.  - small sample size limits generalisability to UK-context. |
| Williams et al. (2023) | UK | 29 adults  Age range:  20 – 50+ years | Focus groups | Exploring public decisions around whether or not to get COVID-19 vaccines, and the facilitators and barriers behind participants’ decisions. | Framework analysis | COVID-19 vaccines | The study identified two main reasons for vaccine delay: a need for more information and waiting until the vaccine was "required." Nine themes emerged, with three facilitators (vaccination as a social norm, necessity, and trust in science) and six barriers (preference for natural immunity, concerns over side effects, lack of information, distrust in government, conspiracy theories, and "COVID echo chambers"). | - qualitative design limits generalisability.  - few participants refused or intended to refuse vaccination.  - older adults were underrepresented.  - changing pandemic conditions may have affected vaccine uptake.  - further research needed to track evolving perceptions. |
| Woodhead et al. (2021) | UK | 25 HCPs and members of senior management  Age range:  *Unclear* | Semi-structured interviews | Understanding the mechanisms underpinning COVID-19 vaccine hesitancy among racial and ethnic minority healthcare staff | Inductive thematic analysis | COVID-19 vaccines | Vaccine decision-making found to be a process of weighing up potential risk and benefits for themselves and for others. | - social desirability might influence the senior management interviews.  - lack of generalisability. |
| Mixed methods design (*n* = 21) | | | | | | | | |
| Bell et al. (2020) | England | 1252 parents & guardians  Age range: 18 -48 years  Interviews with 19 parents & guardians  Age range:  25 – 42 years | Cross-sectional survey & semi-structured interviews | Understanding COVID-19 vaccine acceptability and its reasons. | Thematic analysis | COVID-19 vaccines | Majority of participants would accept vaccines or unsure but leaning towards accepting it which linked to one's trust in vaccines. | - limited generalisability for household income and ethnicity. |
| Bell et al. (2022) | UK | 1917 people surveyed  20 people interviewed  Age range:  35 – 64 years | Cross-sectional surveys & semi-structured interviews | Exploring factors influencing COVID-19 vaccination amongst H&SCWs in the UK | Thematic analysis | COVID-19 vaccines | Reasons for declining vaccination among Black African participants included distrust in COVID-19 vaccination, healthcare providers, and policymakers. | - potential recruitment bias, ethnic groups were not equally represented. |
| Charura et al. (2023) | England (Leeds and surrounds) | 161 unvaccinated adults  *Estimated* age range:  18 – 76 years | Cross-sectional survey including open-ended questions | The study examined lower vaccine uptake in ethnic minority communities, highlighting medical mistrust and perceptions of mattering as key factors influencing hesitancy and vaccination intent. | Thematic analysis | COVID-19 vaccines | Qualitative responses revealed four themes: i) Vaccine seen as a social duty; ii) feelings of pressure and limited autonomy; iii) mistrust rooted in personal and systemic experiences; and iv) concern over restrictions for the unvaccinated. | - cross-sectional design prevents causal conclusions.  - assesses hesitancy and intention, not actual uptake.  - small, non-random sample from one Northern England region.  - limits representativeness and generalisability. |
| Cook et al. (2022) | UK  (Luton) | 1058 residents  Age range:  18 – 65+ years | Survey including open-ended questions | Exploring influential factors that impact the decision to accept the COVID-19 vaccination among an ethnically diverse community. | Content analysis | COVID-19 vaccines | The most common reasons for non-uptake was ‘lack of trust in government/authorities’ and ‘concern of the speed of vaccine development’. | - not generalisable to wider UK population.  - causal interference cannot be established due to cross-sectional nature of the study.  - cultural or historical experiences were not captured. |
| Crawshaw et al. (2024b) | England (London) | 57 migrants  *Estimated* age range:  18 – 55 years | Cross-sectional survey & in-depth interviews, focus groups | Exploring migrant views on delivering catch-up vaccination, including barriers, facilitators, and opportunities. | Deductive, rapid thematic analysis | MMR, tetanus, diphtheria, polio, HPV, & meningitis vaccines | Lack of systems in the UK for catch-up vaccinations upon arrival and emphasized the need for improved healthcare provider skills and knowledge on this issue. | - study affected by resource constraints.  - low recruitment and small sample size limits generalisability to UK migrant population. |
| Davies et al. (2022) | North London borough | 202 pregnant women  Age range:  15 – 45+ years | Cross-sectional survey with open-ended questions | Exploring vaccine uptake, factors associated with vaccination acceptance and underlying beliefs regarding COVID-19 vaccination | Thematic analysis | COVID-19 vaccines | Themes including safety concerns during pregnancy, vaccine effectiveness, and the quality of advice provided. Many unvaccinated women expressed concerns about the vaccine’s safety and its effects on pregnancy, with some feeling inadequately informed. | - small sample size.  - potential self-selection bias and likely under-representation of non-English speakers. |
| Halvorsrud et al. (2023) | England  (London) | 8,310,472 cohort participants  27 London local authority representatives completed qualitative survey  Age range:  *Unclear* | Cohort study with qualitative survey | Understanding system/public health staff’s perceived barriers to vaccination | Thematic analysis | COVID-19 vaccines | Key barriers to vaccine demand included distrust in institutions from systemic racism, information mistrust, particularly among ethnic minorities and women of childbearing age, and financial concerns like unpaid leave. | - interviewees were system leaders/public health staff, not local authority residents.  - younger individuals were not included.  - no real-time data to link interventions with uptake changes.  - confounders like socio-demographic disadvantages were not controlled, often assessed at area level. |
| Husain et al. (2022) | England (Berkshire & Surrey) | 441 pregnant women  Age range:  17 – 44 years | Cross-sectional survey including open-ended questions | Exploring COVID-19 vaccination uptake, facilitators and barriers in ethnically diverse pregnant women. | Thematic analysis | COVID-19 vaccines | Vaccine hesitancy was driven by concerns about the vaccine's effects on the unborn baby and future pregnancies, anxiety about potential adverse impacts on the mother, insufficient knowledge about the vaccine, and a lack of trust in vaccines. | - unreliable response rate data, potential selection bias due to recruitment challenges, limited generalizability to other regions or community-based clinics.  - language barriers affecting ethnic diversity.  - the inability to track when participants were vaccinated in relation to their pregnancy. |
| Husted et al. (2023) | UK | 80 participants (Study 1)  Age range:  18 – 55 years | Multiple studies utilising different methods (Only qualitative study reported) | Identify the factors influencing vaccine uptake. | Framework analysis using Health Belief Model. | COVID-19 vaccines | Vaccine hesitancy was driven by concerns about side effects, particularly fertility risks, and doubts about personal benefit. Barriers to vaccination influenced intentions across all ethnic groups, with BAME communities additionally affected by cues to action. Willingness to vaccinate children declined as their age decreased. | - limited ethnic diversity.  - potential data richness concerns from using surveys over interviews.  - uncertainty in translating intent to behaviour.  - the time-sensitive nature of vaccine beliefs. |
| Jennings et al. (2021) | UK | 1476 adults surveyed  29 adults attended focus groups  Age range:  18 – 87 years | Surveys & focus groups | Exploring the predictors of COVID-19 vaccine hesitancy, | Inductive thematic analysis | COVID-19 vaccines | Vaccine hesitancy is linked to general vaccine and government distrust. Key influences include trust in health experts, perceived threat, herd immunity misconceptions, fears over rapid development and side effects, and beliefs in virus-related conspiracies. Hesitancy is higher among those relying on unregulated social media and holding conspiratorial views. | - self-report of social media sources rather than objective logs.  - small sample size to generalise the findings to the overall country.  - cross-sectional data collection limits understanding causality and change over time. |
| Knight et al. (2023) | England  (Nottingham) | 893 university students  *Estimated* age range:  14 – 27 years | Longitudinal Cohort Study utilising mixed methods survey | Exploring COVID-19 vaccine hesitancy in young adults and whether the reasons behind these attitudes changed during the initial stages of the vaccine rollout. | Thematic analysis | COVID-19 vaccines | The study found that those willing to accept the vaccine primarily cited self-protection, protecting others, and ending the pandemic as key reasons. Hesitancy was mainly driven by a lack of confidence in the vaccine, concerns about insufficient testing, side effects, and long-term risks, which remained consistent across both waves 2 and 3 of data collection. | - limited generalisability due to using a university student sample, which may not reflect broader young adult vaccine acceptance rates.  - 417 participants were lost to follow-up, with non-responders more likely from ethnic minority backgrounds. |
| Mhereeg et al. (2022) | Wales | 331 mothers  Age range:  18 – 50 years | Cross sectional survey including open-ended questions | aimed to (1) assess COVID-19 vaccination rates among pregnant women in Wales by age, ethnicity, and deprivation via EHR data, and (2) explore their views on vaccination during pregnancy using survey responses from the Born-In-Wales Cohort. | Codebook thematic analysis | COVID-19 vaccines | Pregnant women chose vaccination for protection, risk perception, and adequate advice, while hesitancy stemmed from concerns about long-term effects, vaccine anxiety, inconsistent information, and a preference to wait until after pregnancy. | - lack of trimester-specific vaccination data.  - potential selection bias from social media recruitment.  - the use of a convenience sample, limiting generalizability. |
| Neill et al. (2023) | Northern Ireland | 1353 university students & staff  Age range:  18 – 24 years | Cross-sectional survey including open-ended questions | Assessing the level of vaccination and exploring the views of university students and staff on the acceptability of incentives that may encourage uptake. | Thematic analysis | COVID-19 vaccines | Vaccine hesitancy or refusal was due to the perceived lack of research and knowledge of the potential long-term effects at the time of vaccination, religious, personal and ethical beliefs and feeling like vaccinations should not be used to restrict social events, travel and medical challenges. | - potential selection bias from the online survey, and a low response rate (<15%), limiting generalisability.  - As a cross-sectional study from September 2021, it captures a specific moment, restricting causal analysis. |
| Nicholls et al. (2021) | UK | 372 adults  Age range:  65 – 72 years | Cross-sectional survey including open-ended questions | aims to understand at the reasons for older adults not getting the influenza, pneumococcal, and shingles vaccines, | Content analysis | Influenza, pneumococcal & shingles vaccines | Study participants expressed higher Influenza vaccine uptake in comparison to the pneumococcal and shingles vaccines. They also had less awareness on their eligibility for the pneumococcal and shingles vaccines while nearly all participants were aware of Influenza vaccines. | - the qualitative element was based on open-ended questions, which may have limited the depth of understanding. |
| Odejinmi et al. (2022) | England (Sussex) | 378 midwives  Age range:  18 – 60+ years | Mixed methods qualitative and quantitative survey | Understanding midwives’ uptake of and attitudes toward COVID-19 vaccine ethnically diverse groups | Thematic analysis | COVID-19 vaccines | Black midwives had significantly higher levels of vaccine hesitancy compared to White midwives | - selection and representation bias as views limited to those likely to engage with the survey. |
| Roberts et al. (2021) | UK | 4535 people  Age range:  18 – 70+ years | Cross-sectional survey including open-ended questions | Exploring perceptions and attitudes associated with intention to either accept or refuse offers of vaccination in different demographic groups during the early stages of the mass COVID-19 vaccination programme. | multivariate logistic regression, structural text modelling, anthropological assessments, & thematic analysis | COVID-19 vaccines | COVID-19 vaccine hesitancy was primarily driven by concerns about the rapid vaccine development and safety, rather than widespread misinformation. Lower trust in the UK government's decision-making and truthfulness was linked to higher vaccine refusal. Political conservatives were more likely to refuse the vaccine, while liberals were less likely. Those willing to vaccinate cited personal and public protection, with some expressing a sense of collective responsibility. | - non-representative sampling limits generalisability.  Underrepresentation of ethnic minority groups and younger participants.  - self-reported data may introduce bias.  - no causal inferences due to observational design.  - potential influence of confounding variables.  - limited analytical depth from large sample size.  - topic names manually assigned, affecting consistency. |
| Saville et al. (2023) | Wales | 4187 adults  36 interviewed  Age range:  *Unclear* | Cross-sectional survey and semi-structured interviews | providing a rigorous and holistic picture of the challenges facing COVID-19 public health measures in coalmining communities. | Content analysis | COVID-19 vaccines | norms of social solidarity were often invoked by vaccinated respondents, while unvaccinated respondents did not frame decisions in the context of the industrial history of their areas. | - Causation cannot be established due to cross-sectional design. |
| Skirrow et al. (2022) | UK | 1404 survey participants  10 pregnant women interviewed  Age range:  16 – 40+ years | Cross-sectional survey & semi-structured interviews | An online survey and semi-structured interviews were used to investigate pregnant women’s views on COVID-19 vaccine acceptability for themselves when pregnant, not pregnant and for their babies. | Thematic analysis | COVID-19 vaccines | Women expressed common safety concerns about COVID-19 vaccines and general vaccine mistrust, but trust in vaccines and the health system were key factors in their acceptance. | - regional overrepresentation of London and Southeast of England.  -underpowered sample to detect differences in ethnic groups.  - survey was conducted before COVID-19 vaccines were approved, so the results may not reflect changing vaccine attitudes over time. |
| Smith et al. (2023) | UK | 1500 cohort participants  Age range:  18+ | Longitudinal survey in two timepoints (T1 & T2) including open-ended questions | investigated factors associated with COVID-19 vaccine uptake, future vaccination intentions, and changes in beliefs and attitudes over time. | Content analysis | COVID-19 vaccines | At T2, 7.4% remained unvaccinated, with low prior intent, weaker social norms, and concerns about vaccine necessity and safety as key barriers. Those unvaccinated were unlikely to change their minds. | - potential social desirability bias in self-reported data (mitigated by survey anonymity).  - selection bias due to higher vaccination intentions among T2 survey completers.  - no investigation of capability factors (e.g., vaccine access) in logistic regression.  - limited relevance of capability issues during widespread 2021 vaccine rollout. |
| Vilar-Lluch et al. (2023) | England, Wales, & Scotland | 258 adolescents and adults with vaccine hesitancy  Age range:  16 – 24 years | Cross-sectional survey including open-ended questions | Exploring (i) the communication of the vaccination campaign in official UK health messaging, (ii) the vaccination uptake by the UK population and (iii) health message preferences by unvaccinated individuals and vaccine sceptics in order to better promote vaccination among those populations. | Discourse analysis & linguistic corpus analysis | COVID-19 vaccines | Vaccine hesitancy was driven by concerns over side effects, efficacy, and mistrust, with tailored messaging focusing on personal responsibility and prosocial benefits shown to be most effective. | - a non-representative sample, reliance on self-reported data, and a focus on health messaging preferences without considering the broader influence of vaccine efficacy perceptions and institutional trust. |
| Williams et al. (2022) | UK | 423 participants  *Estimated* age range:  18 – 40 years | Cross-sectional survey including open-ended questions | Exploring the factors influence the uptake of regular COVID-19 booster vaccines in the future. | Inductive thematic analysis | COVID-19 vaccines | The main barriers included experiencing side effects with previous COVID-19 vaccines and inaccessibility of vaccination services. Key facilitators included protecting the health of friends and family members, protecting personal health, and maintaining regular activities. | - cross-sectional design limits causal interpretation.  - convenience sampling reduces representativeness.  - demographic skew towards White, younger women.  - focused on intended, not actual, booster uptake.  - vaccine intentions may have shifted as pandemic evolved. |

*Notes.* EHR: Electronic Health Records. H&SCWs: Health and Social Care Workers. HCP: Healthcare Practitioner. HPV: Human Papillomavirus. IMIDs: Immune-Mediated Inflammatory Diseases. MMR: Measles, Mumps, and Rubella. MSM: Men Who Have Sex With Men. NHS: National Health Services. SAGE VH: Scientific Advisory Group of Experts on Immunisation, Vaccine Hesitancy. TDF: Theoretical Domains Framework.

**Estimated* age range: Due to the lack of reported age range data, an estimated age range was derived from summary statistics (e.g., mean, standard deviation) with the assumption of non-normal distribution. This estimate may not reflect the precise age distribution reported in the original studies.

# Appendix 4

***Risk of bias in included studies***

| **Studies** | **Criteria from he Mixed Methods Appraisal Tool** | | | | | | | | | | | | | | | | | | | | | | | | | **Overall quality** |
| --- | --- | --- | --- | --- | --- | --- | --- | --- | --- | --- | --- | --- | --- | --- | --- | --- | --- | --- | --- | --- | --- | --- | --- | --- | --- | --- |
|  | **Qualitative** | | | | | **Quantitative randomised controlled trials** | | | | | **Quantitative non-randomised** | | | | | **Quantitative descriptive** | | | | | **Mixed methods** | | | | |  |
|  | **1.1** | **1.2** | **1.3** | **1.4** | **1.5** | **2.1** | **2.2** | **2.3** | **2.4** | **2.5** | **3.1** | **3.2** | **3.3** | **3.4** | **3.5** | **4.1** | **4.2** | **4.3** | **4.4** | **4.5** | **5.1** | **5.2** | **5.3** | **5.4** | **5.5** |  |
| Anderson et al. (2021) | 1 | 1 | 1 | 1 | 1 |  |  |  |  |  |  |  |  |  |  |  |  |  |  |  |  |  |  |  |  | 100 |
| Bell et al. (2020) | 1 | 1 | 2 | 1 | 1 |  |  |  |  |  |  |  |  |  |  | 1 | 1 | 2 | 2 | 1 | 1 | 1 | 1 | 1 | 1 | 80 |
| Bell et al. (2022) | 1 | 1 | 1 | 1 | 1 |  |  |  |  |  | 1 | 1 | 1 | 0 | 2 |  |  |  |  |  | 1 | 1 | 1 | 1 | 1 | 86 |
| Biesty et al. (2024) | 1 | 1 | 1 | 1 | 1 |  |  |  |  |  |  |  |  |  |  |  |  |  |  |  |  |  |  |  |  | 100 |
| Charura et al. (2023) | 1 | 1 | 2 | 1 | 1 |  |  |  |  |  |  |  |  |  |  | 1 | 1 | 1 | 2 | 1 | 1 | 1 | 1 | 1 | 1 | 86 |
| Cook et al. (2022) | 1 | 0 | 1 | 1 | 1 |  |  |  |  |  | 1 | 1 | 1 | 0 | 1 |  |  |  |  |  | 1 | 1 | 1 | 0 | 1 | 80 |
| Crashaw et al. (2023) | 1 | 1 | 1 | 1 | 1 |  |  |  |  |  |  |  |  |  |  |  |  |  |  |  |  |  |  |  |  | 100 |
| Crashaw et al. (2024a) | 1 | 1 | 1 | 1 | 1 |  |  |  |  |  |  |  |  |  |  |  |  |  |  |  |  |  |  |  |  | 100 |
| Crashaw et al. (2024b) | 1 | 1 | 1 | 1 | 1 |  |  |  |  |  |  |  |  |  |  | 1 | 1 | 1 | 1 | 1 | 1 | 1 | 1 | 2 | 1 | 100 |
| Dasgupta et al. (2024) | 1 | 1 | 1 | 1 | 1 |  |  |  |  |  |  |  |  |  |  |  |  |  |  |  |  |  |  |  |  | 100 |
| Davies et al. (2022) | 1 | 0 | 1 | 1 | 1 |  |  |  |  |  | 1 | 1 | 1 | 0 | 1 |  |  |  |  |  | 1 | 1 | 1 | 1 | 1 | 86 |
| Deal et al. (2021) | 2 | 1 | 2 | 1 | 1 |  |  |  |  |  |  |  |  |  |  |  |  |  |  |  |  |  |  |  |  | 60 |
| Deal et al. (2024) | 1 | 1 | 1 | 1 | 1 |  |  |  |  |  |  |  |  |  |  |  |  |  |  |  |  |  |  |  |  | 100 |
| Denford et al. (2022) | 1 | 1 | 1 | 1 | 1 |  |  |  |  |  |  |  |  |  |  |  |  |  |  |  |  |  |  |  |  | 100 |
| Dennis et al. (2021) | 1 | 1 | 2 | 1 | 1 |  |  |  |  |  |  |  |  |  |  |  |  |  |  |  |  |  |  |  |  | 80 |
| Eberhardt et al. (2023) | 2 | 2 | 1 | 1 | 2 |  |  |  |  |  |  |  |  |  |  |  |  |  |  |  |  |  |  |  |  | 100 |
| Fisher et al. (2021) | 1 | 1 | 1 | 0 | 2 |  |  |  |  |  |  |  |  |  |  |  |  |  |  |  |  |  |  |  |  | 60 |
| Flood et al. (2024) | 1 | 1 | 1 | 1 | 1 |  |  |  |  |  |  |  |  |  |  |  |  |  |  |  |  |  |  |  |  | 100 |
| Friedrich et al. (2024) | 1 | 1 | 1 | 1 | 1 |  |  |  |  |  |  |  |  |  |  |  |  |  |  |  |  |  |  |  |  | 100 |
| Fuller et al. (2022) | 1 | 1 | 1 | 1 | 1 |  |  |  |  |  |  |  |  |  |  |  |  |  |  |  |  |  |  |  |  | 100 |
| Gillibrand et al. (2024) | 1 | 1 | 1 | 1 | 1 |  |  |  |  |  |  |  |  |  |  |  |  |  |  |  |  |  |  |  |  | 100 |
| Gogoi et al. (2022) | 1 | 1 | 1 | 1 | 1 |  |  |  |  |  |  |  |  |  |  |  |  |  |  |  |  |  |  |  |  | 100 |
| Halvorsrud et al. (2023) | 1 | 1 | 1 | 1 | 1 |  |  |  |  |  | 1 | 1 | 1 | 0 | 1 |  |  |  |  |  | 1 | 1 | 1 | 1 | 1 | 93 |
| Hicks et al. (2022) | 1 | 1 | 1 | 1 | 1 |  |  |  |  |  |  |  |  |  |  |  |  |  |  |  |  |  |  |  |  | 100 |
| Husain et al. (2022) | 2 | 1 | 2 | 1 | 1 |  |  |  |  |  |  |  |  |  |  | 2 | 2 | 1 | 1 | 1 | 1 | 1 | 1 | 1 | 2 | 66 |
| Husted et al. (2023) | 1 | 1 | 2 | 1 | 2 |  |  |  |  |  |  |  |  |  |  |  |  |  |  |  |  |  |  |  |  | 60 |
| Jennings et al. (2021) | 1 | 1 | 1 | 1 | 1 |  |  |  |  |  | 1 | 1 | 1 | 0 | 1 |  |  |  |  |  | 1 | 1 | 1 | 1 | 1 | 93 |
| Knight et al. (2023) | 2 | 1 | 2 | 1 | 1 |  |  |  |  |  | 1 | 2 | 1 | 2 | 1 |  |  |  |  |  | 1 | 1 | 1 | 1 | 2 | 66 |
| Lockyer et al. (2021) | 2 | 1 | 1 | 1 | 1 |  |  |  |  |  |  |  |  |  |  |  |  |  |  |  |  |  |  |  |  | 80 |
| Lockyer et al. (2022) | 2 | 1 | 1 | 1 | 1 |  |  |  |  |  |  |  |  |  |  |  |  |  |  |  |  |  |  |  |  | 80 |
| Magee et al. (2022) | 1 | 1 | 1 | 1 | 1 |  |  |  |  |  |  |  |  |  |  |  |  |  |  |  |  |  |  |  |  | 100 |
| Mhereeg et al. (2022) | 1 | 1 | 1 | 1 | 1 |  |  |  |  |  | 1 | 1 | 2 | 2 | 1 |  |  |  |  |  | 1 | 1 | 1 | 1 | 1 | 86 |
| Naqvi et al. (2022) | 1 | 1 | 1 | 1 | 1 |  |  |  |  |  |  |  |  |  |  |  |  |  |  |  |  |  |  |  |  | 100 |
| Neill et al. (2023) | 2 | 1 | 1 | 1 | 1 |  |  |  |  |  |  |  |  |  |  | 1 | 1 | 2 | 2 | 1 | 1 | 1 | 1 | 1 | 2 | 73 |
| Nicholls et al. (2021) | 1 | 1 | 1 | 2 | 1 |  |  |  |  |  |  |  |  |  |  | 2 | 1 | 2 | 2 | 1 | 1 | 1 | 1 | 1 | 2 | 66 |
| Odenjinmi et al. (2022) | 1 | 1 | 0 | 2 | 1 |  |  |  |  |  |  |  |  |  |  | 1 | 0 | 1 | 0 | 1 | 1 | 1 | 1 | 1 | 2 | 66 |
| Ogueji & Okoloba (2022) | 1 | 2 | 2 | 1 | 1 |  |  |  |  |  |  |  |  |  |  |  |  |  |  |  |  |  |  |  |  | 60 |
| Parsons et al. (2024) | 2 | 1 | 2 | 1 | 1 |  |  |  |  |  |  |  |  |  |  |  |  |  |  |  |  |  |  |  |  | 60 |
| Patterson et al. (2023) | 2 | 2 | 1 | 1 | 1 |  |  |  |  |  |  |  |  |  |  |  |  |  |  |  |  |  |  |  |  | 60 |
| Poduval et al. (2023) | 1 | 1 | 1 | 1 | 1 |  |  |  |  |  |  |  |  |  |  |  |  |  |  |  |  |  |  |  |  | 100 |
| Roberts et al. (2021) | 1 | 1 | 1 | 1 | 1 |  |  |  |  |  | 1 | 1 | 1 | 1 | 1 |  |  |  |  |  | 1 | 1 | 1 | 1 | 1 | 100 |
| Saville et al. (2023) | 1 | 1 | 1 | 1 | 1 |  |  |  |  |  | 1 | 1 | 1 | 1 | 1 |  |  |  |  |  | 1 | 1 | 1 | 1 | 1 | 100 |
| Sides et al. (2022) | 2 | 2 | 1 | 1 | 1 |  |  |  |  |  |  |  |  |  |  |  |  |  |  |  |  |  |  |  |  | 60 |
| Skirrow et al. (2022) | 2 | 1 | 2 | 1 | 1 |  |  |  |  |  |  |  |  |  |  | 1 | 1 | 2 | 1 | 1 | 1 | 1 | 1 | 1 | 1 | 80 |
| Smith et al. (2023) | 1 | 1 | 2 | 2 | 1 |  |  |  |  |  | 1 | 1 | 1 | 1 | 1 |  |  |  |  |  | 1 | 1 | 1 | 0 | 2 | 73 |
| Van Bergen et al. (2022) | 1 | 1 | 1 | 1 | 1 |  |  |  |  |  |  |  |  |  |  |  |  |  |  |  |  |  |  |  |  | 100 |
| Vilar-Lluch et al. (2023) | 1 | 1 | 2 | 2 | 1 |  |  |  |  |  | 1 | 1 | 1 | 0 | 1 |  |  |  |  |  | 1 | 1 | 1 | 1 | 1 | 80 |
| Williams et al. (2022) | 1 | 1 | 1 | 1 | 1 |  |  |  |  |  |  |  |  |  |  | 1 | 1 | 2 | 1 | 1 | 1 | 1 | 1 | 1 | 1 | 93 |
| Williams et al. (2023) | 2 | 2 | 1 | 2 | 1 |  |  |  |  |  |  |  |  |  |  |  |  |  |  |  |  |  |  |  |  | 40 |
| Woodhead et al. (2021) | 1 | 1 | 1 | 1 | 1 |  |  |  |  |  |  |  |  |  |  |  |  |  |  |  |  |  |  |  |  | 100 |

*Notes.* 0 = No; 1 = Yes; 2 = Cant tell

1.1: Is the qualitative approach appropriate to answer the research question? 1.2: Are the qualitative data collection methods adequate to address the research question? 1.3: Are the findings adequately derived from the data? 1.4: Is the interpretation of results sufficiently substantiated by data? 1.5: Is there coherence between qualitative data sources, collection, analysis and interpretation? (2.1) Is randomization appropriately performed? (2.2) Are the groups comparable at baseline? (2.3) Are there complete outcome data? (2.4) Are outcome assessors blinded to the intervention provided? 2.5 Did the participants adhere to the assigned intervention? (3.1) Are the participants representative of the target population? (3.2) Are measurements appropriate regarding both the outcome and intervention (or exposure)? (3.3) Are there complete outcome data? (3.4) Are the confounders accounted for in the design and analysis? (3.5) During the study period, is the intervention administered (or exposure occurred) as intended? (4.1) Is the sampling strategy relevant to address the research question? (4.2) Is the sample representative of the target population? (4.3) Are the measurements appropriate? (4.4) Is the risk of nonresponse bias low? (4.5) Is the statistical analysis appropriate to answer the research question? (5.1) Is there an adequate rationale for using a mixed methods design to address the research question? (5.2) Are the different components of the study effectively integrated to answer the research question? (5.3) Are the outputs of the integration of qualitative and quantitative components adequately interpreted? (5.4) Are divergences and inconsistencies between quantitative and qualitative results adequately addressed? (5.5) Do the different components of the study adhere to the quality criteria of each tradition of the methods involved?

# Appendix 5

***Identified themes in the included papers***

| **Theme** | **Subthemes** | **Quotes** | **Papers included** |
| --- | --- | --- | --- |
| ***Contextual Influences*** | | | |
| a. Media environment | Misinformation & conflicting messaging | *“I think it was fear that was driving it (spreading misinformation) to be honest. It was such a difficult time and there were people who were really scared, there was almost like a paranoia.”* (Magee, 2022) | **40**  Bell (2020), Bell (2022), Biesty  (2024), Cook (2022), Dasgupta  (2024), Deal (2021), Deal (2024),  Denford ( 2022), Eberhardt (2023),  Fisher (2021), Flood (2024),  Friedrich (2024), Fuller (2022),  Gillibrand (2024), Gogoi (2022),  Halvorsrud (2023), Hicks (2022),  Husain (2022), Husted (2023),  Jennings (2021), Lockyer (2021),  Lockyer (2022), Magee (2022),  Mhereeg (2022), Naqvi (2022),  Neill (2023), Nicholls (2021), Odejinmi (2022), Ogueji & Okoloba (2022), Parsons (2024),  Patterson (2023), Poduval (2023),  Saville (2023), Sides (2022),  Skirrow (2022), Smith (2023), van  Bergen (2022), Vilar-Lluch (2023),  Williams (2023), Woodhead  (2021) |
|  | Trust & credibility of sources | *“I trust the NHS information however I think the government's message is wrong and misleading. Forcing healthy people to get a vaccine when they wouldn’t end up in hospital and they can still spread it is so wrong!”* (Skirrow, 2022) |  |
|  | Perceived media bias & politicisation of communication | *“All the information I've mainly seen is from like the BBC. So media outlets, or, I think NHS and stuff like that [is like] directly hearing from the government such as Boris*" (Denford, 2022) |  |
|  | Role of social media in spreading  misinformation | *"I see one post saying it has A, B and C and it has this side effect. But*  *I see another post saying oh but this has A, B and C and certain side effects."* (Gillibrand, 2024) |  |
|  | Inadequate, unclear & non-engaging information | *“all of the science has been confusing and changes far too often and doesn’t go far enough to answer why BAME communities are most at risk. I’m not comfortable with the advice being given and the government’s ability to be truthful so I will not be getting a vaccination and I would not want to put my baby at risk either”* (Skirrow, 2022) |  |
|  | Lack of positive media representation | *“if I, it’s on the media that this is really working and, then I’ll be so glad to have one, I don’t mind.”* (Sides, 2022) |  |
|  | Circulating vaccine rumours & conspiracy theories | *“I’ve had my first one and my second one’s tomorrow. I mean, I’m really pleased and I’m really grateful to be getting it, but then you watch on the news and they say about ‘Oh there’s these other variants and the vaccine’s not as strong, ’ then you start thinking ‘oh no, just when you thought you were seeing* *light at the end* *of the tunnel’. . . and you think oh is it going to ever end?”* (P63, AHW, White) (Gogoi, 2022) |  |
|  | Poor-quality Research Papers | *“And some of them are actually not necessarily myths. Some things are based on a couple of scientific reports that then got blown out of proportion. And even though they were disproven after a while, the damage was already done.”* (Poduval, 2023) |  |
| b. Influential figures | Distrust in political figures | *“I think it's too soon/ quick-has [Prime Minister] …had it yet? I think it'll be a while before I get called up so I'll reconsider then.”* (Roberts, 2021)    *“I think just from my experience, there’s a lot of conspiracies that I’ve heard about. Because I mean I identify myself as a Black British person and so within my community I’ve heard a lot of just not trusting the vaccine . . . somebody sent me a video about [UK Government Health Secretary] .. . .suggesting that the vaccine was tested first amongst the BAME group.”* (Williams, 2023) | **16**  Deal (2024), Denford (2022),  Eberhardt (2023), Gillibrand  (2024), Halvorsrud (2023), Husted  (2023), Jennings (2021), Lockyer  (2021), Magee (2022), Naqvi  (2022), Niell (2023), Ogueji &  Okoloba (2022), Roberts (2021),  Smith (2023), Woodhead (2021),  Williams (2023) |
|  | Community leaders building trust | *“I think it’s about [community leaders] taking it themselves. By demonstrating that they’ve had taken it and over time showing them that everything’s okay.”*(Magee, 2022) |  |
|  | Negative reaction to celebrities being part of vaccination campaigns | *“Celebrities are just celebrities, they’re just ordinary people just like you and I. Them taking the vaccine doesn’t mean anything and I can’t prove they’ve actually taken the vaccine.”* (Magee, 2022) |  |
|  | Vaccine ‘influencers’ who spread anti-vaccine conspiracy theories/ pro-vaccine agendas | *“I think [public health messages are] almost trying to scaremonger people and also completely false. I don’t know people my age that have been hospitalised from COVID but I do know people that have been hospitalised from the vaccine. They’re either getting their information very mixed up or someone’s paid them a lot of money to post that”* (Nvx022, not vaccinated). (Denford, 2022) |  |
| c. Historical influences | Historic medical scandals & tragedies | *“Until the vaccine has been properly tested… I am of the thalidomide generation… I would be very wary.”* (Roberts, 2021) | **20**  Anderson (2021), Bell (2022),  Biesty (2024), Charura (2022), Crawshaw (2024a), Dasgupta (2024), Deal (2024), Denford  (2022), Gillibrand (2024), Gogoi  (2022), Halvorsrud (2023), Hicks  (2022), Jennings (2021), Magee  (2022), Ogueji & Okoloba (2022),  Poduval (2023), Roberts (2021),  Saville (2023), Williams (2023),  Woodhead (2021) |
|  | Historical medical testing on ethnic minorities | *“Unethical medical experimentation that has occurred for over a century may be the cause of the fear and mis- trust of doctors and medicines for example... it is difficult to trust medical professionals and their practices peradventure history repeats itself on Ethnic Groups”* (Charura, 2022) |  |
|  | Legacy of vaccine-specific discriminatory practices | *“If you look at things historically, Black and Asian communities have been misused in research […] we have been abused and violated in previous vaccination trials and we can’t deny that.”* (Woodhead, 2021)  *“Interestingly, very common among Afro-Caribbean women, in particular, that they feel that it’s not something for them, because of that problem. And of course, that comes in with the history of vaccination usage over the last century, of how black people were used, for example, as guinea pigs, and I think that inheritance of information, makes people a little bit sceptic and fearful of using the vaccine”* (Dasgupta, 2024) |  |
|  | Distrust stemming from previous pandemics | *“...Sterilization histories and discrimination (e.g., AIDS crisis) and the presence of racism within the healthcare system can affect the rollout of the vaccine”* (Ogueji & Okoloba, 2022) |  |
|  | Historical discrimination & stigmatisation by health systems | *“But if you’re going on outreach in a very deprived community there is often the historical fear that these people take away our kids. And, actually, there’s a massive distrust there about accessing those services”* (Gillibrand, 2024) |  |
| d. Sociodemographic factors | Vaccine distrust among ethnic minorities | *“... I can understand the distrust about medical care from an ethnicity distrust perspective but NOT from an overall anti-vax perspective.”* (Charura 2022)    *"Science[…]doesn't go far enough to answer why BAME communities are most at risk. I'm not comfortable with the advice and the government’s ability to be truthful so I will not be getting a vaccination."* (Skirrow, 2022). | **24**  Bell (2022), Biesty (2024),  Charura (2022), Crawshaw (2024),  Deal (2024), Dasgupta (2024),  Denford (2022), Eberhardt (2023),  Flood (2024), Gillibrand (2024),  Gogoi (2022), Halvorsrud (2023),  Lockyer (2022), Magee (2022), Neill (2023), Ogueji & Okoloba (2022), Poduval (2023), Roberts  (2021), Sides (2022), Skirrow  (2022), Vilar Lluch (2023),  Williams (2022), Williams (2023),  Woodhead (2021) |
|  | Distrust among marginalised  groups | *“[T]here have been issues around confidence and trust in the vaccine*  *and different levels of trust have impacted on differential uptake in vaccine in different groups (e.g., linked to ethnicity, disability)”* (Halvorsrud, 2023) |  |
|  | Religious beliefs & cultural restrictions | “*The first thing is…if its ingredients, like fat and other things, are allowed in Islam. Then, I will research to see how long it would keep me safe…”* (Sides, 2022) |  |
|  | Economic barriers &  inconvenience | *“Actually, the vaccines out there are making lots of money for*  *pharmaceutical companies. And we know, I know, that there is actually far cheaper treatments out there that are equally as effective and actually I really don’t know how effective the vaccines are.”* (Magee, 2022) |  |
|  | Immigration status & access to healthcare | *“A lot of people are afraid, that’s the thing. Once you don’t have that document, so you tend to be afraid to do some certain things. I know some of my, maybe three, friends that have passed away because they are afraid to go to the hospital. They’re afraid to maybe they’re going to ask them some document and all that”* (Deal, 2024) |  |
|  | Age-related concerns | *“You should be open about it [HPV transmission routes]. People our age sort of know. It would be different in the younger year group. But our year, you know more about. . . and it wouldn’t be as sensitive to talk about that with us.”* (Flood, 2024) |  |
|  | Concern for Women’s Health | *“. . .At the time they were like, yes, it’s safe, [Thalidomide], it took them five years to realise the connection. . .So if here we are taking this vaccine during pregnancy . . . but at the same time, we don’t know the long-term effects. So why are you risking it?”* (Magee, 2022)  *“I would get vaccinated if I could trust the vaccine and am not an anti vaccer [sic] but I have concerns about the current vaccine in particular the warning to fertile or pregnant women as well as breastfeeding mothers… I am going to fit into one of these categories for many yrs to come!”*  (Roberts, 2021) |  |
| e. Politics & policies | Distrust in government & political motivations | *“...if the NHS is telling you something you’re more likely to believe it, whereas if it’s a politician, you know, you’ve got much more incentive to think,* ***‘****Well, are they telling the truth?”* (Interviewee #17) (Bell, 2020) | **37**  Bell (2020), Bell (2022), Biesty  (2024), Charura (2022), Cook  (2022), Crawshaw (2024a),  Crawshaw (2024), Dasgupta  (2024), Deal (2021), Deal (2024),  Denford (2022), Eberhardt (2023),  Flood (2024), Gillibrand (2024),  Gogoi (2022), Halvorsrud (2023),  Hicks (2022), Husain (2022),  Husted (2023), Jennings (2021),  Lockyer (2021), Lockyer (2022),  Magee (2022), Mhereeg (2022),  Naqvi (2022), Neill (2023), Ogueji  & Okoloba (2022), Parsons (2024),  Poduval (2023), Roberts (2021),  Saville (2023), Sides (2022),  Skirrow (2022), Smith (2023),  Williams (2022), Williams (2023),  Woodhead (2021) |
|  | Inconsistent & contradictory government messaging | *“And with the government rules, they’re breaking the rules, you know the stories that go around . . .how can you trust a government that keep on breaking the rules?”* (Magee, 2022) |  |
|  | Trusting Health system over government | *“Although I have little faith in the government, I do have faith in the*  *UK's medical approval authorities and processes”* (Roberts, 2021) |  |
|  | Criticism of National Policy | *“Compared to other countries our government has been very inconsistent. One example I think I do have is like maybe New Zealand. And I think it was a Scandinavian country, they were very clear, they were very like, “Well, this is how we’re doing it and this is why we’re doing it.” And they spoke directly to the people. In the UK, it’s the Prime Minister says this, and the health secretary is saying that and they’re just contradicting themselves. So that confuses people.”*  (Poduval, 2023) |  |
|  | Exclusion of communities from policymaking | *“The people in the power, the government, must listen to the voice of*  *all the people antivax. After three months, you must take the vaccine [booster]. It’s no good like that. And then COVID pass is no good.” (Crawshaw 2024a)*  *Before you get to the restaurant, you go to the events like that, you must to show something like that. It’s not work like that. The people now limits. Life, now, is finished.”* (Crawshaw 2024a) |  |
|  | Immigration & legal barriers | *“They should campaign for the free giving of the vaccine without*  *payment. And government should not come and hunt you that you are owing them. Because you took the vaccine you are owing them.”* (Undocumented migrant) (Deal, 2021) |  |
|  | Political influence on health communications | *“I trust science, and our NHS, despite the government's manipulation of the situation.”* (Roberts, 2021) |  |
|  | Pharmaceutical companies granted legal immunity | *“Seeing politicians and the CSO [Chief Scientific Officer] say they would have the vaccine whilst it was still in phase 3 and unapproved destroyed my confidence in it. Pfizer's track record and the fact they have been granted legal immunity also concerns me.”* (Roberts, 2021) |  |
|  | Vaccine Mandating | “*I think my concerns about the vaccine are principally linked to..when the government etcetera imposed a lot of responses which from my point of view are patently disproportionate, it’s difficult to then trust them on any other kind of things they might suggest to tackle it*." (P84,Doctor, Mixed, AD) (Gogoi, 2022) |  |
| f. Social & financial support | Economic barriers | “*Actually, the vaccines out there are making lots of money for pharmaceu- tical companies. And we know, I know, that there is actually far cheaper treatments out there that are equally as effective and actually I really don’t know how effective the vaccines are*.” (Magee, 2022) | **13**  Bell (2020), Biesty (2024),  Crawshaw (2024), Deal (2024),  Gillibrand (2024), Gogoi (2022),  Halvorsrud (2023), Jennings  (2021), Magee (2022), Neill  (2023), Ogueji & Okoloba (2022),  Poduval (2023), Williams (2022) |
|  | Perceived underfunding in vaccine promotion & resourcing | *“I think the government should be spending a lot more money and getting a lot more out there within the communities and making a big effort to give out the proper information. . .there’s communities within communities, and that’s who we have to tackle, because this where the, kind of, in the core, the misinformation starts and it just kind of starts spreading, you know, and so we’ve got to tackle it from the core. ”* (P56, AHP, Asian, AA) (Gogoi, 2022) |  |
|  | Lack of social support following disease-related stigma | *“I saw [mpox messaging] shared from other people, but I saw very little. And with COVID it was everywhere. It was on my timeline from government sources, in terms of government sources had used digital advertising money to put it on my timeline. Which they could have very easily done with monkeypox and targeted towards gay queer bi people.”* (Biesty, 2024) |  |
|  | Inequitable support for marginalised communities | *“You just can’t help but think if we as a BAME community had support in the first place maybe we wouldn’t be the vulnerable ones through COVID.”* (Gillibrand, 2024) |  |
|  | Systematic injustices &  deprivation | *“We feel that for decades, we have been on the receiving end of health and social inequalities. Now, we are being presented with a new vaccine that has been developed in quick time and we are now priority group number 1”* (Bell, 2020) |  |
| ***Group Influences*** | | | |
| a. Family & community experiences with vaccination | Anecdotes raised safety and effectiveness concerns | *“It’s only because you’re hearing this all side effects of vaccines. That was a bit worry. . . I heard from one of my friends who works in a different hospital, one of the people had the vac- cine and they collapsed in less than 3 minutes.”* (Gogoi, 2022) | **28**  Anderson (2021), Bell (2022),  Charura (2022), Crawshaw (2024a), Crawshaw (2024), Deal (2021), Deal (2024), Denford  (2022), Eberhardt (2023), Fisher  (2021), Flood (2024), Friedrich  (2023), Gillibrand (2024), Gogoi  (2022), Halvorsrud (2023), Hicks  (2022), Lockyer (2021), Lockyer  (2022), Magee (2022), Naqvi  (2022), Patterson (2023), Poduval  (2023), Roberts (2021), Saville  (2023), Sides (2022), Smith  (2023), Williams (2023),  Woodhead (2021) |
|  | Personal stories of death or illness of loved ones | *“Friends that have been done, my family, one of them already got injected and she had a complain with fever, with fatigues, maybe at two weeks’ time is (sic). And she was like, ‘Please, just take a minute before you just take’.”* (Hicks 2022) |  |
|  | Family and community as trusted sources | *“My family and community, I take advice from. It's a cultural thing. If I have a headache, they're the people I ask first. Even with the vaccination, I take a lot of advice from my Caliph, my community is a go-to place.”* (Eberhardt, 2023) |  |
|  | Shared experiences in marginalised communities | *“I think just from my experience, there’s a lot of conspiracies that I’ve heard about. Because I mean I identify myself as a Black British person and so within my community I’ve heard a lot of just not trusting the vaccine . . . somebody sent me a video about [UK Government Health Secretary] Matt Hancock . . .suggesting that the vaccine was tested first amongst the BAME group.”* (Williams, 2023) |  |
|  | Influence of pregnancy and maternal networks | *“I have a friend that got really... she got ME and narcolepsy from the swine flu vaccine, because that was developed pretty quickly I think as well, and I think that’s just stuck with me”* (Pregnant woman)  (Anderson, 2021) |  |
|  | Social Isolation of Vaccine Hesitant Individuals | *“Participant L started distributing information to her vaccine-hesitant WhatsApp group, for instance, after feeling invalidated for sharing her concerns about the vaccine with her family. Providing another illustration of how information sharing is entwined with social support [75], the emphasis on inclusion and exclusion also raises questions about the role that pooling plays in helping people to mediate stigma and other barriers to engagement.”* (Hicks, 2022) |  |
| b. Significant others’ beliefs,  attitudes about health & prevention | Family as the primary source of influence | *“My husband is completely anti vac so…I’d be in a bit of a difficult position to try and get my children vaccinated”* (Sides, 2022)  *“When we talked to them [resident women], they said “look, our fathers are frightened that no one will marry us if we take the vaccine”* (Halvorsrud, 2023) | **22**  Biesty (2024), Deal (2024),  Denford (2022), Eberhardt (2023),  Flood (2024), Gillibrand (2024),  Gogoi (2022), Halvorsrud (2023),  Hicks (2022), Husain (2022),  Lockyer (2021), Lockyer (2022),  Magee (2022), Naqvi (2022), Neill  (2023), Parsons (2024), Patterson  (2023), Sides (2022), smith (2023),  van Bergen (2022), Williams (2023), Woodhead (2021) |
|  | Community as a trusted source over authorities | “*This is very much a nationally driven programme and it doesn’t work locally. You need local figures, local influence, understanding of the local situation, and then you need to flex your programme locally.”* (Halvorsrud, 2023) |  |
|  | Social perceptions and stigma around vaccination | *“Because I remember I was getting a lot of abuse online because I posted quite a lot about it [mpox]. Like, saying look if you’re affected by this, my heart goes out. And I got a lot of trolls... [saying] you deserve it, thinking that I had the disease myself.”* (Biesty, 2024) |  |
|  | Interpersonal pressure and disengagement | *“the pressure to take that vaccine has become so much because we don’t want to be blamed. It has no longer become a choice. It has become it’s you against them; if you’re not with us you’re against us. And that’s the pressure the BAME community is fac- ing more and more...”* (Gillibrand, 2024) |  |
|  | Guidance from the workplace | *“Initially when it [vaccine] came out a lot of the [front line-workers] on my team were like ‘nope, not going to get it’ but that changed one-by-one, they started to go for it, and now I think there is only a handful who haven’t gone for it . . . its being monitored by senior management but they have got to ask have you had it or not, they are not asking why you haven’t had it, but I don’t know if that is something at some point, you know you have to justify your grounds as to why you haven’t”* (Williams, 2023) |  |
| c. Experiences with health systems | Healthcare professionals as trusted but inconsistent sources | *“I once went to see the GP because I had some spots on my back, and I was prescribed steroids, and it made the problem worse and I still have the spots now. That makes me think they don’t know me and at my age I know my body and what I react to.”* (Naqvi, 2022).  *“Was difficult to get advice on what to do early on….Midwife said she wouldn’t get it”.* (Davies, 2022) | **31**  Anderson (2021), Bell (2022),  Biesty (2024), Charura (2022),  Cook (2022), Crawshaw (2024), Crawshaw (2024a), Crawshaw (2024b), Davies (2022), Deal (2021), Deal (2024), Denford  (2022), Eberhardt (2023), Flood  (2024), Gillibrand (2024), Gogoi  (2022), Hicks (2022), Husain  (2022), Lockyer (2021), Magee  (2022), Naqvi (2022), Neill  (2023), Ogueji & Okoloba (2022),  Parsons (2024), Patterson (2023),  Poduval (2023), Roberts (2021),  Saville (2023), Sides (2022),  Skirrow (2022), Woodhead (2021) |
|  | Distrust of government and pharmaceutical industry | *“No confidence or trust whatsoever in the testing regimes for the vaccines. If they are so good why has [the] government given immunity from prosecution in the event of adverse reactions to big pharma companies?”(*Roberts, 2021) |  |
|  | Lack of cultural sensitivity and marginalisation | *“when you’ve got structures like the council and the NHS, how they are perceived in the community; it’s like us and them... You know, particularly around councils, you know, they are trying to do outreach. But if you’re going on outreach in a very deprived community there is often the historical fear that these people take away our kids. And, actually, there’s a massive distrust there about accessing those services”* (Gillibrand, 2024) |  |
|  | The role of schools and healthcare in vaccine education | *“(when asked why there is a lack of vaccine education) ‘Teachers are maybe scared to talk about it because of the repercussions in the school. So it’s not really individual teachers’ fault. It’s the fault of higher up. . . because it is a Catholic school and teachers are maybe scared to give you that information, and the correct information.”* (S13, Focus group 3; Female only Catholic school) (Flood, 2024) |  |
|  | Access and availability of health services | *“There were national outlets saying vaccines are available, […] then they call the clinic and clinic say we're not 100% sure what were actually doing with it yet..It just breaks down the trust a little bit between people and services”* (Biesty, 2024) |  |
|  | Disengagement with Health systems | *“You’d have to tie me down and strap me down to put a vaccine in me…I don’t do medicine, I don’t do doctors, I don’t do clinics anyway.”* (Sides, 2022) |  |
|  | Unaddressed longstanding health inequalities | *“There has been a lot of mistrust among the community, for example some of the communities will be asking why suddenly is NHS interested in the Black and Asian minority ethnic group? Over years we have spoken, they’ve never listened to us, what has changed now, what is the intention here?”* (P59, Doctor, Black, AA) (Gogoi, 2022) |  |
| d. Immunisation as a social  norm | Social pressure to vaccinate  without behaviour change | *“I’m only doing it because the government seem to be saying that they won’t let me travel without it ...feeling pressured to take it. I have I’ve been forced into taking because of my profession.”* (Charura 2022) | **21**  Bell (2022), Charura (2022), Crawshaw (2024a), Deal (2021), Deal (2024), Fisher (2021), Flood  (2024), Gillibrand (2024), Hicks  (2022), Jennings (2021), Lockyer  (2022), Nicholls (2021), Odejinmi  (2022), Patterson (2023), Poduval  (2023), Roberts (2021), Saville  (2023), Sides (2022), smith (2023),  Williams (2023), Woodhead  (2021) |
|  | Variability in immunisation norms across communities | *“There’s about three or four of us, sometimes. We’re like little old ladies, standing on the landing, talking about all sorts of issues, and the COVID and the vaccination and, you know? And I think, out of us, only one’s had it. You know? And the rest of them has the similar feeling that I’ve got, you know what I mean? We just talk about the same thing and, the way I say I feel, that’s them, as well.”* (Poduval, 2023) |  |
|  | Religious norms against vaccination | *“Some people think, “Oh, if I’m going to die of COVID, I might as well take the vaccine and get less side-effects from it, from the severe form of the virus, and then at least I won’t die.” But whether you’re going to die or you’re not going to die, as a Muslim we leave that in the hands of God. So God decides who’s going to die and who’s going to live. So you know, it’s not in our hands. So you could take the double-jab, get COVID, get it severe and pass away. It can, it can happen. It’s happened to family members.”* (Poduval, 2023) |  |
|  | Feelings of force, mandates and coercion | *"I had no option, I had to do it, and also everybody else had done it”* (Gillibrand et al, 2024). |  |
|  | Assertion of personal choice | *“I’m still holding out. I’ve been sent several letters and loads of text messages telling me that I needed to go for the vaccination, but I would rather wait at least four months to see exactly where it is all going. . . I don’t want to be sort of pressured into something that I might regret”* (Williams, 2023) |  |
|  | Collective feeling of not being vaccinated | Participant L started distributing information to her vaccine-hesitant WhatsApp group, for instance, after feeling invalidated for sharing her concerns about the vaccine with her family. (Hicks, 2022) |  |
|  | Herd immunity as a myth | *“Because herd immunity is essentially a myth: measles, TB, polio, smallpox- none of these (and others) were ever eliminated from a population by herd immunity [presumably meaning by uncontrolled natural spread of infection], but by vaccination.”* (Roberts, 2021) |  |
| ***Vaccine-specific Influences*** | | | |
| a. Risk/benefit (evidence based) | Lack of long-term evidence and safety concerns | *“Not enough evidence to say it would work, no evidence to say it doesn’t cause side effects.”* (Skirrow, 2022) | **37**  Anderson (2021), Bell (2020),  Bell (2022), Biesty (2024),  Charura (2022), Crawshaw (2023),  Dasgupta (2024), Deal (2024),  Denford (2022), Eberhardt (2023),  Fisher (2021), Fuller (2022),  Gillibrand (2024), Gogoi (2022),  Hicks (2022), Husain (2022),  Knight (2023), Lockyer (2021),  Lockyer (2022), Magee (2022),  Naqvi (2022), Neill (2023),  Nicholls (2021), Odejinmi (2022), Ogueji & Okoloba (2022), Parsons (2024), Patterson (2023), Poduval  (2023), Roberts (2021), Roberts  (2021), Saville (2023), Skirrow  (2022), Sides (2022), Smith (2023), Williams (2022), Williams  (2023), Woodhead (2021) |
|  | Concerns about vaccine efficacy, effectiveness | *“The first thing is…if its ingredients, like fat and other things, are*  *allowed in Islam. Then, I will research to see how long it would keep me safe…”* (Sides, 2022) |  |
|  | Reports of minor side effects and daily life impact | *“...Yes, I received the first dose, and you know what? My hand was sore the day after. I am scared because I do not want to get sick from this sore...”* (Ogueji & Okoloba, 2022) |  |
|  | Reports of serious side effects (e.g. blood clots) | *“My issue was on the blood clot side because when I had my kid, I was bleeding a lot, I lost 1 litre plus. So, when I heard on the news that people were having blood clots I said, my God, it makes me feel really scared.”* (P5, female) (Crawshaw, 2023) |  |
|  | Negative experiences with previous vaccinations | *“My son had flu when he was four and a half, and ended up in hospital for a couple of nights”* (Anderson, 2021). |  |
| b. Vaccine novelty | Perceived insufficient testing and speed of development | *“If I’m honest, I was concerned, because of the fact that they’d produced a vaccine so quickly.”* (Gogoi (2022)    *“Not enough testing (8–12 months compared to 8–12 years for normal vaccine development), too much secrecy regarding content and method of production, problems arising even in early cases.”* (Roberts, 2021) | **38**  Anderson (2021), Bell (2020),  Bell (2022), Charura (2022), Cook  (2022), Crawshaw (2023), Davies  (2022), Deal (2021), Denford  (2022), Eberhardt (2023), Fisher  (2021), Gillibrand (2024), Gogoi  (2022), Husain (2022), Husted  (2023), Jennings (2021), Knight  (2023), Lockyer (2021), Lockyer  (2022), Magee (2022), Mhereeg  (2022), Naqvi (2022), Neill  (2023), Nicholls (2021), Odejinmi  (2022), Ogueji & Okoloba (2022),  Parsons (2024), Patterson (2023),  Roberts (2021), Saville (2023),  Sides (2022), Skirrow (2022),  Smith (2023), van Bergen (2022),  Vilar-Lluch (2023), Williams  (2022), Williams (2023),  Woodhead (2021) |
|  | Preference to delay vaccination until more information | *“you know, when you sort of heard things in the news like well, it’s been 18 years and they haven’t developed a SARS vaccines, or whatever it was that was sort of banded around yesterday, it sort of makes you go, why is that? So, I think that if a vaccine was to come out like this one could do in a hurry it need to have associated information to how this has been able to happen so quickly.”* (Interviewee 19)- (Bell, 2020) |  |
|  | Lack of long-term data and Unknown future harms | *“For me, it’s the long term of not knowing what it can do in the future. Although it can work now, would it have any effects on me in five years?... Just certain things have not been answered for me, so that’s why I’ve opted not”* (Denford 2022) |  |
|  | Uncertainty about vaccine  effectiveness | *“ I doubted it […] it’s insane that the vaccine can be produced in such a short time […] is this vaccine even going to work?”* (Eberhardt, 2023) |  |
|  | Fear of specific side effects linked to novelty | *“Right now, I am going to refuse it, until the very last moment. I feel like I’m a guinea pig. I don’t know if you heard the news that they have stopped one of the vaccines because there has [sic] been cases of blood clots of something. I know it’s a very, very, very, very tiny per-centage but I feel like if I wait till the very last more [information] can come out”.* (Williams, 2023) |  |
|  | Preference to delay vaccination until more information | *“I want to personally wait […] until the testing had been fully done and then I was gonna make a decision you know just by being cautious.”* (Dennis, 2021) |  |
| c. Mode of administration | Fear of needles and injection anxiety | *“Have a severe phobia of needles, trying to get over it though”* (Neill, 2023) | **12**  Crawshaw (2023), Crawshaw (2024a), Deal (2024), Flood (2024), Gillibrand (2024), Husain  (2022), Neill (2023), Nicholls  (2021), Parson (2024), Poduval  (2023), Sides (2022), Williams  (2023) |
|  | Distrust in the booster concept | “*The people in the power, the government, must listen to the voice of all the people antivax. After three months, you must take the vaccine [booster]. It’s no good like that It’s not work like that. The people now limits. Life, now, is finished.”* (Crawshaw, 2024a) |  |
|  | Preferences for vaccination settings | *“There was general agreement across ethnic minority and white British groups on preferred places to receive the vaccine, including community healthcare settings and settings perceived as low risk, for example, a space with less people.”* (Sides, 2022) |  |
|  | Transparency in the vaccination process | *“More information and transparency on how the vaccines have been developed. There seems to be many categories of anti-vaccers, ranging from conspiracy theories to people who are genuinely worried that vaccines may have long- term currently unknown side effects.”* (Neill, 2023)  *Government and medics need to have an informed information campaign rather than just ‘get vaccinated’ message.”* (Neill, 2023) |  |
| d. Vaccination programs | Inconsistent public health messaging regarding eligibility | *“Information was almost released to the public before local systems had had a chance to catch up with it...there were national outlets saying vaccines are available, speak to your local clinic...I feel like it does more harm than good if I’m doing all my out- reach and speaking with loads of queer people in Manchester and saying oh vaccines are available, and then they call the clinic and clinic say actually we’re not 100% sure what we’re doing with it yet...It just breaks down the trust a little bit between people and services.”* (Biesty, 2024) | **19**  Bell (2022), Biesty (2024),  Crawshaw (2024), Deal (2024),  Denford (2022), Eberhardt (2023),  Gogoi (2022), Halvorsrud (2023),  Husted (2023), Magee (2022),  Naqvi (2022), Neill (2023), Ogueji & Okoloba (2022), Patterson (2023), Poduval (2023), Roberts  (2021), Saville (2023), Sides  (2022), Woodhead (2021) |
|  | Lack of representation and ethical allocation concerns | *“It very much reflects the white gaze (throw a bunch of ‘BAME [Black, Asian, and Minority Ethnic]’ people together as though they are not from separate and het- erogenous communities, appeal to racial stereotypes (e.g. Indian doctors), push the desired narrative with a script of ‘facts’ and ‘debunking’ rather than encouraging genuine dialogue, etc.). It felt like the way the NHS tries to appeal to us, there is no effort to actually understand and address the reasons for our reluctance/ refusal and thus came across as patronizing/inauthentic...speaking at us rather than to us.”* (Eberhardt, 2023) |  |
|  | Ineffective vaccination program strategies | *“But it was okay and then when I went there it was a little bit scary because the police and the National Guard and people were there to help line up cars, as there was a different type of situation than I’ve ever seen before. Like when I got my other vaccines there were no police, no National Guard there, you know, it was just going to a regular doctor’s appointment, this was different and it felt different.”* (Saville, 2023) |  |
|  | Previous mass immunisation campaigns | *“When I was back home, they can use one needle to treat many people. I don’t know if they stopped it. But here, when they use one needle, you will see them put it in the bin. They don’t repeat it again for any other person”* (Deal, 2024) |  |
|  | Nationally driven programs (rather than local) | *‘This is very much a nationally driven programme and it doesn’t work locally. You need local figures, local influence, understanding of the local situation, and then you need to flex your programme locally.”* (Halvorsrud, 2023) |  |
| e. Reliability of vaccine supply source | Distrust in the Pharmaceutical Industry | *“Actually, the vaccines out there are making lots of money for pharmaceutical companies. And we know, I know, that there is actually far cheaper treatments out there that are equally as effective and actually I really don’t know how effective the vaccines are.”* (Magee, 2022) | **25**  Bell (2020), Biesty (2024),  Charura (2022), Cook (2022), Deal  (2024), Eberhardt (2023), Gogoi  (2022), Husain (2022), Husted  (2023), Jennings (2021), Lockyer  (2021), Magee (2022), Naqvi  (2022), Neill (2023), Nicholls  (2021), Odejinmi (2022), Ogueji &  Okoloba (2022), Patterson (2023),  Poduval (2023), Roberts (2021),  Sides (2022), Skirrow (2022), van Bergen (2022), Williams (2023),  Woodhead (2021) |
|  | Perceived lack of credibility or inclusivity in testing | *“…reassurance that any vaccines and trials have been completed on BAME individuals…health is set up with perhaps white British people in mind.*” (Sides, 2022) |  |
|  | Ethical concerns about vaccine development practices | *“Vaccines developed using aborted foetal cells are unethical. If there is a vaccine that has been developed without using aborted foetal cells in any part of the manufacture or testing processes, I would be happy to receive that vaccine.”* (Roberts 2021) |  |
|  | Concerns about storage and efficacy | *“I probably won't get this until near the end of 2021 or the beginning of*  *2022. This slow rollout of the vaccination could be for many reasons e.g., staff availability; the number of vaccines procured; problems with storage/transportation, etc.”* (Ogueji & Okoloba, 2022) |  |
|  | Vaccine ingredients | *“The first thing is…if its ingredients, like fat and other things, are allowed in Islam. Then, I will research to see how long it would keep me safe…”* (Sides, 2022) |  |
| f. Vaccination schedule | Concerns over booster frequency | Participants shared their frustrations about changes to the interval between vaccine doses, against the manufacturers recommended protocol, and for some this negatively affected COVID-19 vaccination confidence and left them feeling vulnerable. Several participants felt the dosing interval change was not well communicated. (Bell, 2022) | **10**  Bell (2020), Bell (2022), Deal  (2024), Eberhardt (2023), Gogoi  (2022), Husain (2022), Parsons  (2024), Roberts (2021), Sides  (2022), Williams (2022) |
|  | Uncertainty about duration of protection | *“I would like to see the result of studies into how long antibodies last post vaccination and also learn more about how people are reacting to it. So my answer is NO I won't take it as soon as it becomes available. By the time my turn comes my answer might well be YES.”* (Roberts, 2021) |  |
|  | Vaccine overload and necessity of routine vaccines | *“The unvaccinated are being told that they must be vaccinated to be protected but the vaccinated are being told they are not fully protected and need a "booster". For this reason, […] I choose not to have a vaccine as I have no faith"* (Husain, 2022) |  |
| g. Healthcare provider knowledge & attitudes | Inconsistent guidance from healthcare professionals | *“I’ve heard it from both sides, I’ve heard it from a GP that is saying no, don’t get it because you know your life is at risk here is the information, and I’ve heard it from a [hospital doctor’s] point of view saying ‘no you need to have it, this is the reasons why’, so I’ve had it from both sides and I’m more saying no, but you know when you just feel pressured into it, I think I’m kind of more leaning into being pressured into getting the injections.”* (Saville 2023) | **26**  Charura (2022), Crawshaw (2024a), Crawshaw (2024b), Davies (2022), Deal (2021), Deal  (2024), Denford (2022), Flood  (2024), Fuller (2022), Gogoi  (2022), Hicks (2022), Husain  (2022), Lockyer (2021), Mhereeg (2022), Naqvi (2022), Neill  (2023), Odejinmi (2022), Ogueji &  Okoloba (2022), Patterson (2023),  Roberts (2021), Saville (2023),  Sides (2022), Skirrow (2022),  smith (2023), Woodhead (2021) |
|  | Disagreements among scientists and vaccine developers | *“If you look at scientists, [..], they all have different opinions and none of those opinions are factual…[…] So when I look at these stats, I think to myself, well, is this true?”* (Denford, 2022) |  |
|  | Influence of trusted healthcare providers | *“My midwife told me not to get it. GP also said no and Pharmacy…”* (Davies, 2022) |  |
|  | Poor communication from healthcare providers | *“... Had they said, ’Yes, you should take it, these are the reasons why, this is the research,’ then I probably would have”* (Patterson, 2023) |  |
|  | Knowledge Gaps in healthcare | “*And I don’t think your average GP. they’ve got a medical background, but it doesn’t make them an expert in a new emerging virus that no-one’s met before.”* (Hicks, 2022) |  |
|  | Perception of bias and discrimination | *“Do not trust these medical professionals.”* (Charura 2022) |  |
| ***Perceptual Influences*** | | | |
| a. Trust | Distrust in government motives, messages and health authorities | *“I just feel very let down by the government that had they been very... I feel they haven’t been honest all along and I think I’m not the only one who feels that. I think majority of the UK population feels that, that the government has never been very honest about anything to do with COVID...I can’t trust anything the government says. Because they’ll say one thing, then they’ll do a U-turn and say something else”* (Gillibrand, 2024) | **42**  Bell (2020), Bell (2022), Bell  (2022), Biesty (2024), Charura  (2022), Cook (2022), Crawshaw  (2024), Crawshaw (2024a), Crawshaw (2024b), Dasgupta (2024), Deal (2021), Deal (2024),  Denford (2022), Eberhardt (2023),  Flood (2024), Gillibrand (2024),  Gogoi (2022), Halvorsrud (2023),  Hicks (2022), Husain (2022),  Husted (2022), Jennings (2021),  Lockyer (2021), Lockyer (2022),  Magee (2022), Naqvi (2022), Neill  (2023), Nicholls (2021), Odejinmi  (2022), Ogueji & Okoloba (2022), Parsons (2024), Patterson (2023),  Poduval (2023), Roberts (2021),  Saville (2023), Sides (2022),  Skirrow (2022), Smith (2023), Vilar Lluch (2023), Williams (2022), Williams (2023),  Woodhead (2021) |
|  | Distrust in Pharmaceutical companies, Science and the Medical Profession | “*You’d have to tie me down and strap me down to put a vaccine in me…I don’t do medicine, I don’t do doctors, I don’t do clinics anyway*.” (Sides, 2022). |  |
|  | Distrust in vaccine promotion and incentives | *“there’s the trust issue with this, how much they want us to know. They want us to go back to work, they want people back in offices, they want people back on trains and on buses, because that economically is important to the government*” (Gillibrand, 2024) |  |
|  | Differing trust in healthcare providers vs government | *“although I don’t trust the ... government, I do trust the scientists that have worked on the vaccine. If they say it’s safe, then I shall accept that”* (Husted, 2022) |  |
|  | Trust built through early interactions with the health system | *“[i]t’s not something that’s going to go away, and we’re not going to ... shift that those ... Those that mistrust for quite some time. So I think we have to be honest with ourselves ... why people, you know, find it difficult to say, yeah, I’ll get my vaccination”* (Halvorsrud, 2023) |  |
| b. Emotions (fear) | Fear of side effects and injections | “*Have a severe phobia of needles, trying to get over it though*”. (Neill, 2023) | **37**  Anderson (2021), Bell (2020),  Bell (2022), Biesty (2024),  Charura (2022), Crawshaw (2024a), Dasgupta (2024), Deal (2021), Deal (2024), Denford  (2022), Eberhardt (2023), Flood  (2024), Friedrich (2023),  Gillibrand (2024), Hicks (2022),  Husain (2022), Jennings (2021),  Lockyer (2021), Lockyer (2022),  Magee (2022), Mhereeg (2022),  Neill (2023), Nicholls (2021), Odejinmi (2022), Ogueji & Okoloba (2022), Parsons (2024),  Patterson (2023), Poduval (2023),  Saville (2023), Sides (2022),  Skirrow (2022), smith (2023), van  Bergen (2022), Vilar Lluch (2023),  Williams (2022), Williams (2023),  Woodhead (2021) |
|  | Confusion and scepticism over necessity and efficacy | *“I think it is very confusing. The things they have on official websites, social media talks about it, and you don’t know what to trust.”* (Mhereeg, 2022) |  |
|  | Anger and Frustration toward societal pressure | *“I am seeing a lot of people every day stating they will not be taking the vaccine, none of these people have any qualifications or experience in healthcare, they are just simply scared, and angry at their government’s response to the pandemic. Governments need to explain in colloquial language what the vaccine is and how it will help.”* (Ogueji & Okoloba (2022). |  |
|  | Worry about specific health outcomes | “*Basically I don’t want to die and I don’t want my husband to die and I don’t want my baby to come out weird.”* (Anderson, 2021) |  |
|  | Fear from 'Scare-Tactic' vaccine messaging | “*I think [public health messages are] almost trying to scaremonger people and also completely false. I don’t know people my age that have been hospitalised from COVID but I do know people that have been hospitalised from the vaccine.*” (Denford, 2022) |  |
|  | Lack of entitlement and fear of health authorities | *"The prime minister has said we want everybody to be vaccinated. But he did not say no information will be given to the home office. He did not give that safety blanket and security to people that are afraid to give information away. So, this is my fear -Undocumented migrant”* (Deal, 2021) |  |
|  | Anxiety and Uncertainty about vaccine safety | *“I think it was fear that was driving it (spreading misinformation) to be honest. It was such a difficult time and there were people who were really scared, there was almost like a paranoia.”* (Magee, 2022). |  |
|  | Emotional self-protection from vaccines | *“I have removed the news from my feed, so I don’t have them appearing on my feed, but then every couple of days I still go check the news, but I just, I’m more in charge of deciding what I want to read, versus just seeing a scary headline appearing on my news- feed on my phone*.” (Participant K) (Hicks, 2022) |  |
|  | Guilt and regret from societal pressure | *"I think it’s the anxiety and actually guilt- I think one of the biggest things there is guilt [...] I feel kind of trapped, like if I don’t do it I’m potentially going to catch COVID which would also put my child at risk, but if I do it, I could potentially cause her to have lifelong or even fatal consequences"* (Denford, 2022) |  |
| c. Preferences | Preferences for visible evidence of vaccine safety | *“I would like to know more about side effects. Besides what is online there appears to be a vagueness about the side effects that I’m sure some people have had. I think there should be more transparency especially for Black and brown healthcare workers.”* (Bell, 2022) | **21**  Bell (2020), (Bell, 2022), Biesty  (2024), Crawshaw (2023),  Crawshaw (2024), Crawshaw (2024b), Deal (2021), Denford (2022), Flood (2024), Mhereeg  (2022), Neill (2023), Nicholls  (2021), Odejinmi (2022), Patterson  (2023), Roberts (2021), (Saville,  2023), Sides (2022), van Bergen  (2022), Williams (2022), Williams  (2023), Woodhead (2021) |
|  | Alternative 'natural' and 'less risky' prevention strategies | *“I put something into my mouth only when it becomes too hurtful. I always delay it to the last moment. Like a high temperature or whatever pain, I try to cope myself… So, we use all the sources of our bodies first before taking something from outside"* (Deal, 2021) |  |
|  | Convenience, accessibility, and low frequency of boosters | *“Dont think its necessary considering already having 2 jabs”* (Williams, 2022) |  |
|  | Preferences for health risks over side effects | *“While I’m while I’m given the choice, my, my preference and choice is to say no, thank you, I’ll take my, I’ll take my riskI’m not gonna get vaccinated just ’cause someone else thinks I should, you know, as long as they don’t try and impose it on me, or, or, honestly, try and convince me. You know it’s live and let be. And if that if that means I die, well, so be it, I die, that was my choice.”* (Saville, 2023) |  |
|  | Communication and vaccine-learning style preferences | *“Option of remote learning. I intend to be vaccinated; however, I am concerned about my young family with a 3-year-old and newborn and that the vaccine does not necessarily protect you from contracting COVID-19. The university cannot guarantee every student or member of staff to be vaccinated and spread of the virus may still occur”* (Neill, 2023). |  |
|  | Preferences for other vaccines (e.g. flu) | *“I prefer flu vaccine because that one will protect you”* (Crawshaw, 2023)    *“Now I’ve had it I feel okay about it, but I think that’s because with the Pfizer it doesn’t seem to be any negative reports, whereas with AstraZeneca there seems* *to be a lot of mixed communications, and I don’t think there is a lot of fault with the vaccine, it’s just I don’t think the company is very good at kind of being truthful and that makes people a bit doubtful . . . I don’t think I would have done it [had the AstraZeneca vaccine]”* (Participant 24, Female, 50+). (Williams, 2023) |  |
| d. Prosocial behaviour | Prioritisation of vulnerable, at-risk and older populations | *“Those people who were shielding and those people who have a higher chance of being affected by it, should get it first… So those people over 50 should get it first.”* (Sides, 2022) | **15**  Bell (2020), Bell (2022),  Dasgupta (2024), Deal (2021),  Denford (2022), Fisher (2021),  Hicks (2022), Husain (2022),  Husted (2022), Lockyer (2022),  Neill (2023), Roberts (2021), Sides  (2022), van Bergen (2022),  Williams (2023) |
|  | Questioning the vaccines role in public safety | *“Or if I see somebody is just trying to manipulate other people because they don’t have [unintelligible] I will just pop in and give a few facts and leave.”* (Hicks, 2022) |  |
|  | Hesitancy from doubts of the prosocial benefits of COVID-19 vaccine | *“As a healthy young person, it is statistically proven that the vaccine is more likely to cause me harm than the virus is. Also, you can carry the virus regardless of whether or not you’re vaccinated so for me to be vaccinated would not impact anyone else’s safety. These two points together are the reasons I will not be vaccinated”.* (Neill, 2023) |  |
| e. Health-related beliefs | Low perceived risk of illness (e.g. COVID-19, flu, HPV) | *“I’m quite healthy so I’d hold back, I wouldn’t go straight away.”* (sides, 2022) | **34**  Anderson (2021), Bell (2020),  Bell (2022), Biesty (2024), Deal  (2024), Denford (2022), Eberhardt (2023), Fisher (2021), Flood  (2024), Fuller (2022), Gogoi  (2022), Hicks (2022), Husain  (2022), Husted (2023), Jennings  (2021), Lockyer (2021), Lockyer  (2022), Mhereeg (2022), Naqvi  (2022), Neill (2023), Odejinmi  (2022), Ogueji & Okoloba (2022),  Parsons (2024), Patterson (2023),  Poduval (2023), Roberts (2021),  Saville (2023), Sides (2022), Smith  (2023), van Bergen (2022), Vilar  Lluch (2023), Williams (2022),  Williams (2023), Woodhead  (2021) |
|  | Low perceived severity of disease | *"My own immune system is a better way to fight it off, given that I am a healthy, fit person and have not needed flu vaccines I have no need for this. I do not believe it is as big a threat as it has been made out”* (Neill, 2023) |  |
|  | Low perceived susceptibility to disease due to sociodemographic factors | *“It doesn’t stop you catching it or spreading it—it only potentially reduces the symptoms and as a young fit healthy woman, I’d rather not take the risk of injecting the unknown into my body.”* (Husain, 2022) |  |
|  | Health optimism and risk-benefit analysis | *“I suppose myself I think I had sort of, like, few of the risk factors personally. . . so I don’t think that I’ve ever felt particularly that I was at risk of becoming detrimentally unwell for my own health. . .[but] the fear of the unknown I suppose and I guess you’re putting your own health at risk, you’re putting that of anybody that you live with at risk, I was always scared about going to work and catching the virus and then passing it on. . .* ” (P8, Doctor, White, AA) (Gogoi, 2022) |  |
|  | Concerns for fertility and maternal health | “*Yeah, I worry enough about having a miscarriage or something like that just naturally, so I think adding a potential further risk factor would make me think twice.”* (Anderson, 2021) |  |
|  | Conspiracy theory beliefs | *“I think it makes me a lot less likely to get a vaccine, because then it makes me more paranoid about the conspiracy theories that I hear, and well, why are they forcing so many people to get it if they don’t want to get it?... Yeah, I wouldn’t agree with that, I think there’ll be a huge uprising if they did that*” (Nvx019, not vaccinated). (Denford, 2022) |  |
|  | Novelty of COVID-19 | “*There’s a concern that, if one takes the vaccine and a variant of a new variant emerges that could be resistant to the vaccine then there’s a question of do you have to keep taking vac- cines to protect yourself against each single variant? I don’t really have any intention of getting the vaccine*.” (Williams, 2023) |  |
| f. Vaccine-specific beliefs | High perceived risk of vaccination | *“Risks associated with negative side effects from the vaccine outweigh the risks associated with the virus”* (Husted, 2023) | **46**  Anderson (2021), Bell (2020),  Bell (2022), Biesty (2024), Charura (2022), Cook (2022),  Crawshaw (2024), Crawshaw (2024b), Davies (2022), Deal (2024), Denford (2022), Eberhardt  (2023), Fisher (2021), Flood  (2024), Friedrich (2023), Fuller  (2022), Gillibrand (2024), (Gogoi,  2022), Halvorsrud (2023), Hicks  (2022), Husain (2022), Husted  (2023), Jennings (2021), Knight  (2023), Lockyer (2021), Lockyer  (2022), Magee (2022), Mhereeg  (2022), Naqvi (2022), Neill  (2023), Nicholls (2021), Odejinmi  (2022), Ogueji & Okoloba (2022),  Parsons (2024), Patterson (2023),  Poduval (2023), Roberts (2021),  Saville (2023), Sides (2022),  Skirrow (2022), Smith (2023), van  Bergen (2022), Vilar Lluch (2023),  Williams (2022), Williams (2023),  Woodhead (2021) |
|  | Low perceived effectiveness and necessity | “*Not sure whether effective or not, I know people have had it and still fallen ill”* (Davies, 2022) |  |
|  | Risk-benefit analysis and personal  health | “*Risks associated with negative side effects from the vaccine outweigh*  *the risks associated with the virus*” (Husted, 2023)    *“I have allergies. . .I do worry about having the vaccine when I seem to have all, exacerbated my allergies. . .I do have concerns. . .my [family member] has had [adverse reactions] following some previous immunisation. . .and with my allergies [and health conditions], I am fearful that I might end up having [similar adverse reactions]. . .and so yeah, so I have got some fears about vaccination.* ” (P76, Nurse/Midwife, Mixed). (Gogoi, 2022) |  |
|  | Distrust in vaccine safety and government motives | *“I’m not comfortable with the advice being given and the government’s ability to be truthful so I will not be getting a vaccination and I would not want to put my baby at risk either”* (Skirrow, 2022) |  |
|  | Preference for natural immunity | *“I’m not taking vaccine because, you know, the second wave I didn’t got COVID, I’ve got my natural immunisations, so I’m not really fancying the vaccine either.”* (Gogoi, 2022) |  |
|  | Moral, ethical and religious beliefs | *“Elderly individuals…they have nothing to lose…I will ask or request my mother to have it, considering by balancing or weighing the risks and the advantages. But not for the young individuals, not for the children.”* (Sides, 2022) |  |
|  | Vaccine conspiracy theorists | *“I think just from my experience, there’s a lot of conspiracies that I’ve heard about. Because I mean I identify myself as a Black British person and so within my community I’ve heard a lot of just not trusting the vaccine . . . somebody sent me a video about [UK Government Health Secretary] …. . .suggesting that the vaccine was tested first amongst the BAME group.”* (Williams, 2023) |  |
|  | General Negative Attitudes towards Vaccines | “*Unless my life is really, really at risk, I will wait a long time before I take that vaccine because I’m not really fan of vaccines per se*” (Sides, 2022) |  |
| h. Personal sensitivity to vaccine | Pregnancy-related sensitivity and concerns | “*I have had my first vaccine, albeit slightly delayed, I definitely did question whether I was comfortable to have the vaccine, not from a race perspective so much as from a fertility per- spective I was concerned.”* (Gogoi, 2022) | **30**  Anderson (2021), Bell (2022),  Dasgupta (2024), Davies (2022),  Deal (2024), Denford (2022),  Flood (2024), Fuller (2022),  Gillibrand (2024), Gogoi (2022),  Hicks (2022), Husain (2022),  Husted (2023), Lockyer (2022),  Magee (2022), Naqvi (2022), Neill  (2023), Nicholls (2021), Odejinmi  (2022), Ogueji & Okoloba (2022),  Parsons (2024), Patterson (2023),  Poduval (2023), Roberts (2021),  Saville (2023), Sides (2022), smith  (2023), van Bergen (2022),  Williams (2022), Woodhead  (2021) |
|  | Experiences of adverse reactions and side effects | *“I get quite anxious in those kind of environments and I haven’t reacted very well when I’ve had vaccines before*.” (Participant W) (Hicks, 2022) |  |
|  | Sensitivity due to medical conditions | *“I do have concerns. . .and with my allergies [and health conditions], I am fearful that I might end up having [similar adverse reactions]...and so yeah, so I have got some fears about vaccination.”* (Gogoi, 2022) |  |
|  | Needle phobia and psychological sensitivity | *“Have a severe phobia of needles, trying to get over it though*”. (Neill, 2023) |  |
|  | Perceived discrimination and lack of personalised care | *“it would influence people more to get it because it’s really recommended for you, not just, ’We think you should do this.’ If it’s there in writing on your wee checklist, you’re like, ’Right, okay, I need to get that done.”* (Patterson, 2023) |  |
|  | Religious concerns | *“A lot of people um use kind of religious reasons for not taking it. Um‘cause there was something in the news today about some of the components of the vaccine is um, genes from kind of unborn babies or something crazy like that so, a lot of religious people will not take the vaccines for those sorts of reasons.”* (Woodhead, 2021) |  |
|  | Age-related concerns | *“I don’t know if I was convinced. Yes, I trusted it. Trusted it as much as they could be trusted. I mean, I’m [in my 60s], I’m not a youngster. Perhaps down the line, there may be an enduring side effect. Who knows? I don’t know. Nobody knows. We’re allowed to have doubts, aren’t we?*” (Saville, 2023) |  |
| ***Practical Abilities*** | | | |
| a. Memory | Poor memory of past vaccines leading to unfamiliarity | *“Yeah, I mean, I’ve had a little bit of ... I mean, I think it’s natural to have a little bit of anxiety that ... I remember the, after, like, one of the... But obviously, I’ve never, I’ve never had a flu vaccine outside of pregnancy, ’cause I’m not eligible otherwise”* (Parsons, 2024) | **15**  Bell (2020), Crawshaw (2024a), Deal (2021), Fisher (2021), Flood  (2024), Friedrich (2023),  Gillibrand (2024), Jennings  (2021), Magee (2022), Naqvi  (2022), Parsons (2024), Roberts  (2021), Saville (2023), Sides  (2022), Vilar Lluch (2023) |
|  | Ineffective vaccine communication and low retention | *“You go to school and you learn about these things and then you just forget it when you go home. But when you see it on social media it reminds you, and you’re like, oh yeah!”* (Focus group 1; Female only Catholic school) (Flood, 2024) |  |
|  | Preference for small, interactive learning environments | *“Students recalled external facilitators who did practical activities and indicated that they remembered most from these enjoyable interactive classes”* (Flood, 2024) |  |
|  | Memory of past injustices and  racism increasing hesitancy | “*It’s a lifetime of experience, [], you’re waiting for the outcomes and nothing happens, yet some people are sleeping under the arches and others have got three or four houses. []...as you can tell, I’m not into injustices”* (community resident, female, 75–79, Black Caribbean). (Gillibrand, 2024) |  |
| b. Knowledge | Lack of vaccine-specific knowledge | *"So I haven’t had the vaccine yet. Mainly because, obviously information”* (Gillibrand et al, 2024) | **45**  Anderson (2021), Bell (2020),  Bell (2022), Biesty (2024),  Charura (2022), Cook (2022),  Crawshaw (2024), Crawshaw (2024a), Dasgupta (2024), Deal (2021), Deal (2024), Denford  (2022), Eberhardt (2023), Fisher  (2021), Flood (2024), Fuller  (2022), Gillibrand (2024), Gogoi  (2022), Hicks (2022), Husain  (2022), Husted (2023), Jennings  (2021), Knight (2023), Lockyer  (2021), Lockyer (2022), Magee  (2022), Mhereeg (2022), Naqvi  (2022), Neill (2023), Nicholls  (2021), Odejinmi (2022), Ogueji & Okoloba (2022), Parsons (2024),  Patterson (2023), Poduval (2023),  Roberts (2021), Saville (2023),  Sides (2022), Skirrow (2022),  smith (2023), van Bergen (2022), Vilar Lluch (2023), Williams (2022), Williams (2023),  Woodhead (2021) |
|  | The need for clear, relevant scientific research. | *“...Accurate information needs to be provided on the vaccine, who is making these vaccines, and how relia- ble the pharmaceutical companies are (Why have they not mentioned a lot about this? And does this vaccine mean we definitely won't get COVID-19 even if it is a new variant?”* (Ogueji, 2021) |  |
|  | Uncertainty around vaccine safety and risks | *“There is no actual facts on how safe this vaccine is during pregnancy. One minute your told pregnant women should not take it then they should […] Issues will arise in time to come”* (Husain, 2022) |  |
|  | Poor access to trusted sources of information | “*They can’t tell me whether I need to have–I can’t–they gave me my vaccine [first dose] and they can’t answer, like, I can’t find somebody to ask the question, questions I need answers for about my second vaccine. I can’t find the right person to talk to.* ” (P86, AHP, Other, PA) (Gogoi, 2022) |  |
|  | Misconceptions, misinformation and myths | *“For me, I would like to take the vaccine if that will make everything better. But the fake news is scaring me, so I don’t know. That is a problem. I don’t know if it’s real, I don’t know if it’s fake. When you take it, it will change the DNA… it will stop the person not having kids in future. A lot of stories are flying”* (Deal 2021) |  |
|  | Poor awareness on specific vaccines mechanisms | *“I don’t think [the vaccine] is working very well, and the reason why I say that is because I don’t think it’s a vaccine. And I’m not medically trained or anything like that, but in my opinion, a vaccine is something that stops you from contracting something. And the Coronavirus vaccine does not stop you from con- tracting it, it actually stops the effects of it, doesn’t it? Or lessens the effects of it”* (Nvx020, not vaccinated). (Denford, 2022) |  |
|  | Importance of Informed Choice | *“I think what would have benefitted in my case was having the leaflet, the vaccination leaflet, before I had the vaccine rather than being given that after I’ve had it because it included a lot of information that people could benefit from, that I could have benefitted, and I didn’t have to go fact finding and I wouldn’t have had that doubt if I knew exactly what to expect and how it worked and what it was. ”* (P58, AHP, Asian, PA) (Gogoi, 2022) |  |
| c. Resources | Financial barriers to vaccine access | “*I have to travel far to receive my vaccines which is difficult with my small income and travel abilities"* (Williams, 2022). | **20**  Anderson (2021), Bell (2022), Biesty (2024), Crawshaw (2024a), Crawshaw (2024b), Deal (2021), Deal (2024), Flood (2024), Fuller  (2022), Gogoi (2022), Halvorsrud  (2023), Husted (2023), Neill  (2023), Ogueji & Okoloba (2022),  Parsons (2024), Patterson (2023),  Sides (2022), Smith (2023), van  Bergen (2022), Williams (2022) |
|  | Inequities in Vaccine Delivery and  Access | *“Some survey responders reported they would be more inclined to receive the COVID-19 vaccine in “pop-up vaccination clinics in spaces accessible to younger people who may not be able to drive/have their own car”.”* (Neill, 2023) |  |
|  | Transportation and transport barriers | *"It depends on the distance. Because as I said, we are not able to offer ourself to transport, we cannot pay for it. At some points like some people they have health issue where they cannot walk so long. I mean long distance. So, it depends on the distance and in some conditions maybe it would be in the GP, in some others it would be the [walk-in vaccination] centre”* (Deal, 2021) |  |
|  | Appointment and booking inaccessibility | “*My optimism about actually having that available within the next 18 months is quite low*.” (Sides, 2022) |  |
|  | Supply shortages and underresourcing | *“Information was almost released to the public before local systems had had a chance to catch up with it...there were national outlets saying vaccines are available, speak to your local clinic...I feel like it does more harm than good if I’m doing all my out- reach and speaking with loads of queer people in Manchester and saying oh vaccines are available, and then they call the clinic and clinic say actually we’re not 100% sure what we’re doing with it yet...It just breaks down the trust a little bit between people and services.”* (Biesty, 2024) |  |
|  | Educational resources and Access in schools | *“There’s no education into vaccines, even at school there’s nothing. I suppose if you have that continuing education, it’s something you can make an informed decision*” (Patterson, 2023) |  |
| d. Capability | Physical barriers and health  conditions | “*If we go again [to] the GP in this country, when you try to phone them, maybe have to take you two hours, three hours, before to pick up the phone. And then when they pick up the phone in reception, okay, they give you now appointment. Appointment may be two weeks or three weeks before to see you. I don’t know. [. . .] Is embarrassing. For now, the GP, I think, is a joker. [. . .] When they come to see you after that, they give you a paracetamol. What’s going on? We work in this country. We pay the tax. . . Why the thing must go like that? Why?”* (Crawshaw, 2024a) | **25**  Anderson (2021), Biesty (2024), Crawshaw (2024a), Crawshaw (2024b), Dasgupta (2024), Deal (2021), Deal (2024), Denford  (2022), Flood (2024), Friedrich  (2023), Fuller (2022), Gillibrand  (2024), Gogoi (2022), Halvorsrud  (2023), Lockyer (2021), Neill  (2023), Odejinmi (2022), Ogueji &  Okoloba (2022), Parsons (2024),  Patterson (2023), Sides (2022),  Smith (2023), van Bergen (2022),  Williams (2022), Woodhead  (2021) |
|  | Occupational and scheduling barriers | *“I didn’t actually get my whooping cough until I was, like, 30 weeks, because I wasn’t actually offered it. They told me to get it, but the only way I could get it was through the doctor. And then to book an appointment, you know, sitting on the phone at eight o’clock in the morning with everybody else was just, no. So you’d think that would be, that they’d be able to book you in for that”* (Parsons, 2024) |  |
|  | Language and system navigation barriers | *“But if you’re going to get somebody else who doesn’t look like you, doesn’t sound like you, doesn’t speak your language; that trust is not there, necessarily”.* (Woodhead, 2021) |  |
|  | Cognitive and psychological barriers | *“The information that’s given is not user friendly. . .you can’t hand that to a young person and expect them to read through that. . .maybe our parents can read through that but I think it would need to be edited down for us to be able to understand that.*’(S13, Focus group 3; Female only Catholic school) (Flood, 2024) |  |
|  | Lack of autonomy and parental influence | *“...forcing us. It’s not recommending now. Enforcement. If you don’t get it you cannot live your life in England now because of that vaccine.”* (Gillibrand, 2024) |  |
|  | Exclusion from Health Systems | *“Some of the asylum-seekers and the refugees, they don’t have a GP, so I don’t know how the government will help out with that. If the government can speak with the charities, because a lot of these refugees and asylum seekers, they use different charities"* (Deal, 2021) |  |
| e. Organisation | Perceived inconvenience of vaccination | *“It is an inconvenience*” (Williams, 2022) | **12**  Biesty (2024), Crawshaw (2024b), Deal (2021), Gogoi (2022), Neill  (2023), Ogueji & Okoloba (2022),  Parsons (2024), Patterson (2023),  Sides (2022), Smith (2023),  Williams (2022), Williams (2023) |
|  | Short notice and poor planning  from authorities | *“Information was almost released to the public before local systems had had a chance to catch up with it...there were national outlets saying vaccines are available, speak to your local clinic...I feel like it does more harm than good if I’m doing all my out- reach and speaking with loads of queer people in Manchester and saying oh vaccines are available, and then they call the clinic and clinic say actually we’re not 100% sure what we’re doing with it yet...It just breaks down the trust a little bit between people and services.”* (Biesty, 2024) |  |
|  | Limited appointment and follow-up time | *“I didn’t actually get my whooping cough until I was, like, 30 weeks, because I wasn’t actually offered it. They told me to get it, but the only way I could get it was through the doctor. And then to book an appointment, you know, sitting on the phone at eight o’clock in the morning with everybody else was just, no. So you’d think that would be, that they’d be able to book you in for that”* (P3, 25 –34, White British). (Parsons, 2024) |  |

*A subtheme that fits into multiple themes.

# Appendix 6

***Vaccine Hesitancy Across the Lifespan: Adolescence, Adulthood, and Older Adulthood***

|  | **Studies** | **Themes /Subthemes** |
| --- | --- | --- |
| **Adolescence**  12-18 years  (*n* =2) | Fisher et al. (2021)  Flood et al. (2024) | SAGE Framework  *Contextual Influences*  Media environment; Sociodemographic factors; Politics and Policies  *Group Influences*  Family and Community experiences with vaccination; Significant others’ beliefs, attitudes about health and prevention; Experiences with health systems  *Vaccine-specific influences*  Mode of administration; Healthcare provider knowledge and attitudes  PaPA Framework  *Perceptual Influences*  Trust; Emotions; Preferences; Immunisation as a social norm; Risk/Benefit (Evidence based)  Vaccine Novelty; Prosocial behaviour; Health-Related Beliefs; Vaccine-Specific Beliefs; Personal Sensitivity to Vaccine  *Practical Influences*  Memory; Knowledge; Resources; Capability |
| **Adulthood**  18 - 64 years  (*n* = 19) | *Qualitative studies:*  Anderson et al. (2021), Biesty et al. (2024), Deal et al. (2021), Denford et al. (2022), Dennis et al. (2021), Gogoi et al. (2022), Husted et al. (2023), Parsons et al. (2024), Patterson et al. (2023), Sides et al. (2022), Van Bergen et al. (2022)  *Mixed methods studies:*  Bell et al. (2020), Bell et al. (2022), Crawshaw et al. (2024b), Husain et al. (2022), Husted et al. (2023), Mhereeg et al. (2022), Neill et al. (2023), Williams et al. (2022) | *All themes covered.* |
| **Older adulthood**  65+ years  (*n* = 1) | Nicholls et al. (2021) | SAGE Framework  *Contextual Influences*  Media Environment  *Group Influences*  Immunisation as a social norm  *Vaccine-specific influences*  Risk/Benefit (Evidence Based); Vaccine Novelty; Mode of Administration; Reliability of Vaccine Supply Source  PaPA Framework  *Perceptual Influences*  Trust; Emotions; Preferences; Vaccine-Specific Beliefs; Personal Sensitivity to Vaccine  *Practical Influences*  Knowledge |

*Note.* Mixed age groups were excluded from the comparative analysis. Mixed age groups either examined adults and older adults together (*n* = 21) or adolescents and adults (*n* = 2) jointly. Some papers did not specify age ranges (*n* = 6).
